# Productive visualization of high-throughput sequencing data using the SeqCode open portable platform

**DOI:** 10.1038/s41598-021-98889-7

**Published:** 2021-10-01

**Authors:** Enrique Blanco, Mar González-Ramírez, Luciano Di Croce

**Affiliations:** 1 Centre for Genomic Regulation (CRG), Barcelona Institute for Science and Technology (BIST), Dr. Aiguader 88, 08003 Barcelona, Spain; 2 Universitat Pompeu Fabra (UPF), Barcelona, Spain; 3 ICREA, Passeig Lluis Companys 23, 08010 Barcelona, Spain

**Keywords:** Computational biology and bioinformatics, Data integration, Data processing

## Abstract

Large-scale sequencing techniques to chart genomes are entirely consolidated. Stable computational methods to perform primary tasks such as quality control, read mapping, peak calling, and counting are likewise available. However, there is a lack of uniform standards for graphical data mining, which is also of central importance. To fill this gap, we developed SeqCode, an open suite of applications that analyzes sequencing data in an elegant but efficient manner. Our software is a portable resource written in ANSI C that can be expected to work for almost all genomes in any computational configuration. Furthermore, we offer a user-friendly front-end web server that integrates SeqCode functions with other graphical analysis tools. Our analysis and visualization toolkit represents a significant improvement in terms of performance and usability as compare to other existing programs. Thus, SeqCode has the potential to become a key multipurpose instrument for high-throughput professional analysis; further, it provides an extremely useful open educational platform for the world-wide scientific community. SeqCode website is hosted at http://ldicrocelab.crg.eu, and the source code is freely distributed at https://github.com/eblancoga/seqcode.

## Introduction

Current high-throughput sequencing techniques (e.g. ChIP-seq, ATAC-seq, and RNA-seq) can use a single run to identify the repertoire of functional characteristics of the genome. Therefore, an accurate interpretation of the results is fundamental to understand how the transcriptomic and epigenomic landscape of cells evolve during developmental and/or disease stages^1–3^. Powerful bioinformatic tools are available to manage this volume of data at a primary stage: (i) quality control profilers evaluate distinct scoring metrics on raw information^4–6^; (ii) mapping algorithms identify the location of each read on the genome^7–9^; (iii) peak callers find clusters of reads significantly enriched in certain genomic regions in the sample map file^10–12^; (iv) genome browsers are useful to visualize genome-wide binding profiles and peaks^13–16^; and (v) other auxiliary applications convert intermediate files into the appropriate data formats^17–20^.

However, further work is necessary to finally extract useful knowledge from processed data (such as meta-plots, heatmaps, feature charts, boxplots of signal intensity, etc.). Surprisingly, only moderate interest has been shown until now for standardizing the design of this kind of graphical information. As a result, researchers in many cases develop their own scripts to address these issues, which is likely to compromise reproducibility and comparisons. In parallel, the reduction in the cost of the next-generation sequencing (NGS) techniques has dramatically increased the number of high-throughput sequencing experiments that are produced every year, making it urgent to find methods that efficiently process this information^21^.

Here, we first illustrate the main characteristics of SeqCode, introduce the collection of principal SeqCode features to perform high-quality graphical analysis of sequencing data, and propose a standardized nomenclature of representations. SeqCode is entirely focused on the graphical analysis of 1D genomic data (e.g. ChIP-seq, RNA-seq). For information regarding visualization of 3D data (such as Hi-C interactions), we refer the reader to existing comprehensive reviews^22–25^. Next, we discuss our web portal, which offers a user-friendly interface for SeqCode functions to generate images from NGS data sets. From technical and biological point of views, we then assess the soundness of SeqCode results in several realistic scenarios. Finally, we comprehensively review the existing literature on similar tools to evaluate our software in comparison to current approaches.

## Results

### Overview of the SeqCode software

**Figure 1:**
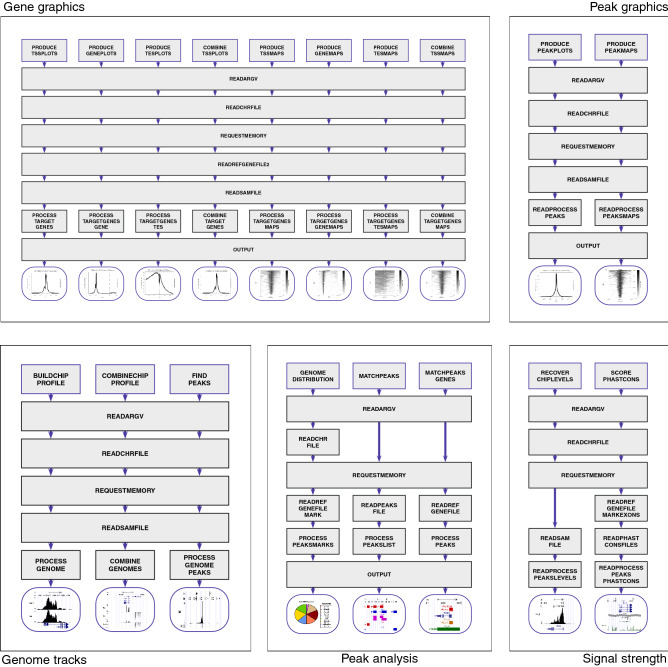
Modular architecture of SeqCode. Diagram of subroutines implemented in SeqCode to perform multiple tasks. Functions were classified depending on the object of analysis: genes, genomic regions or peaks, genome tracks, and signal levels. Functions are represented as boxes; arrows indicate the dataflow of each pipeline.

SeqCode is a command-line toolkit that produces top-quality images from high-throughput sequencing data. It has been implemented in ANSI C following a modular architecture of blocks (Fig. 1). All commands share a common standard UNIX interface, which makes our software a suitable candidate for integration into most bioinformatic pipelines. Each command is configurable, allowing the user to customize multiple attributes for the final plots (Table 1 for a comprehensive list of functions). SeqCode is fully functional over almost every genome that has been made available to scientific community. Information on a particular genome assembly is loaded from two external files that must be supplied by the user: (i) the chromosome size file (ChromInfo.txt), and (ii) the gene transcript annotations (refGene.txt), as provided by the RefSeq consortium^26^. Both archives can be downloaded from the UCSC genome browser for every genome release^14^. Moreover, users must specify the filename containing the reads mapped over the same genome assembly in SAM/BAM format^18^. Each analysis is usually performed across a list of target genes or a subset of genomic intervals—structured as BED records—that are also provided by the user within a plain text file. SeqCode output typically consists of graphical summaries of a sequencing experiment for the fraction of genes or genomic regions selected by the user. SeqCode tools routinely normalize data by sequencing depth (i.e. total read count in the experiment), although users can opt for performing the normalization by spike-in correction (i.e. total read count in the exogenous material). R is internally used to generate the resulting images to PDF. Intermediate plain text files and R scripts of each run are also delivered into the output folder labelled with the name of the job.

**Table 1:** List of SeqCode functions.

	Name	Description	Input	Output
Custom tracks for genome browsers	buildChIPprofile	Generates a custom track from a sequencing experiment to be visualized in current genome browsers	One SAM/BAM file	The custom track in BedGraph format
	combineChIPprofiles	Generates a custom track by subtracting the second sequencing experiment from the first one to be visualized in current genome browsers	Two SAM/BAM files	
Average occupancy plots	combineTSSplots	Draws the average distribution by subtracting the second sequencing experiment from the first one around the TSS of selected genes	Two SAM/BAM files, a list of genes	The average plot in PDF, the signal values and the R script
	produceGENEplots	Draws the average distribution of a sequencing experiment along the body of the meta-gene of selected genes	One SAM/BAM file, a list of genes	
	producePEAKplots	Draws the average distribution of a sequencing experiment around the center of selected peaks	One SAM/BAM file, a list of BED peaks	
	produceTESplots	Draws the average distribution of a sequencing experiment around the TES of selected genes	One SAM/BAM file, a list of genes	
	produceTSSplots	Draws the average distribution of a sequencing experiment around the TSS of selected genes	One SAM/BAM file, a list of genes	
Density heatmaps	combineTSSmaps	Draws the heatmap by subtracting the second experiment from the first one around the TSS of selected genes	Two SAM/BAM files, a list of genes	The heat map in PDF, the rank of signal values and the R script
	produceGENEmaps	Draws the heatmap of reads of an experiment along the body of the meta-gene of selected genes	One SAM/BAM file, a list of genes	
	producePEAKmaps	Draws the heatmap of reads of an experiment around the centre of selected peaks	One SAM/BAM file, a list of BED peaks	
	produceTESmaps	Draws the heatmap of a sequencing experiment around the TES of selected genes	One SAM/BAM file, a list of genes	
	produceTSSmaps	Draws the heatmap of a sequencing experiment around the TSS of selected genes	One SAM/BAM file, a list of genes	
Signal levels	recoverChIPlevels	Calculates the average, maximum, and total number of normalized reads of a sequencing experiment inside a set of regions of the genome	One SAM/BAM file, a list of regions in BED format	Average, maximum and total number of reads inside each region
Peak analysis	genomeDistribution	Distributes a list of regions of the genome into distinct gene features	A list of regions in BED format	The genome distribution in PDF, the annotation of the regions
	matchpeaks	Calculates the overlap between the components of two lists of peaks	Two lists of BED peaks	The list of overlapping peaks and the subsets of peaks that do not overlap on the other set
	matchpeaksgenes	Identifies genes genome-wide that contain one (or more) peaks from a list of selected peaks defined by the user accordingly to a set of rules and distances	A list of BED peaks	The list of genes in the genome that are target of the peaks
Evolutionary conservation	scorePhastCons	Calculates the average, maximum value, and total PhastCons score inside a set of genomic regions	A list of regions in BED format	Average, maximum and total score inside each region

SeqCode can be executed in UNIX platforms under heterogeneous processor and memory configurations. We have tested SeqCode on iMac (Mac OS-X) and Personal Computer (Ubuntu Linux) platforms. The full source code is distributed upon open GNU license in GitHub (https://github.com/eblancoga/seqcode). We provide a Makefile to generate the binaries from the standalone version, which can be immediately used in the working path of the user machine. Users can automatically check that SeqCode services are functioning correctly with fragments of published sequencing samples using a collection of Perl scripts that is integrated into the code distribution. Moreover, we provide binary files for UNIX platforms, Oracle VM virtualbox™ appliances and Docker containers built on Linux Ubuntu, for different memory and storage configurations with the latest version of SeqCode internally pre-installed, through our website (http://ldicrocelab.crg.eu/). Our interactive web platform also offers front-end graphical interfaces implemented in PHP for SeqCode core functions, and other auxiliary tools designed in R, to generate graphical representations of user datasets (e.g. boxplots, heatmaps, PCA plots, scatter plots, UpSet charts^27^, alluvial charts, and Venn diagrams).

### Components of SeqCode

SeqCode can generate quantitative and qualitative graphical representations, thereby allowing novel biological knowledge to be extracted from sequencing data. We briefly introduce each component of our suite of programs (Table 1; see our website for further information).

### Custom profiles for quick visualization of fresh data in genome browsers

**Figure 2:**
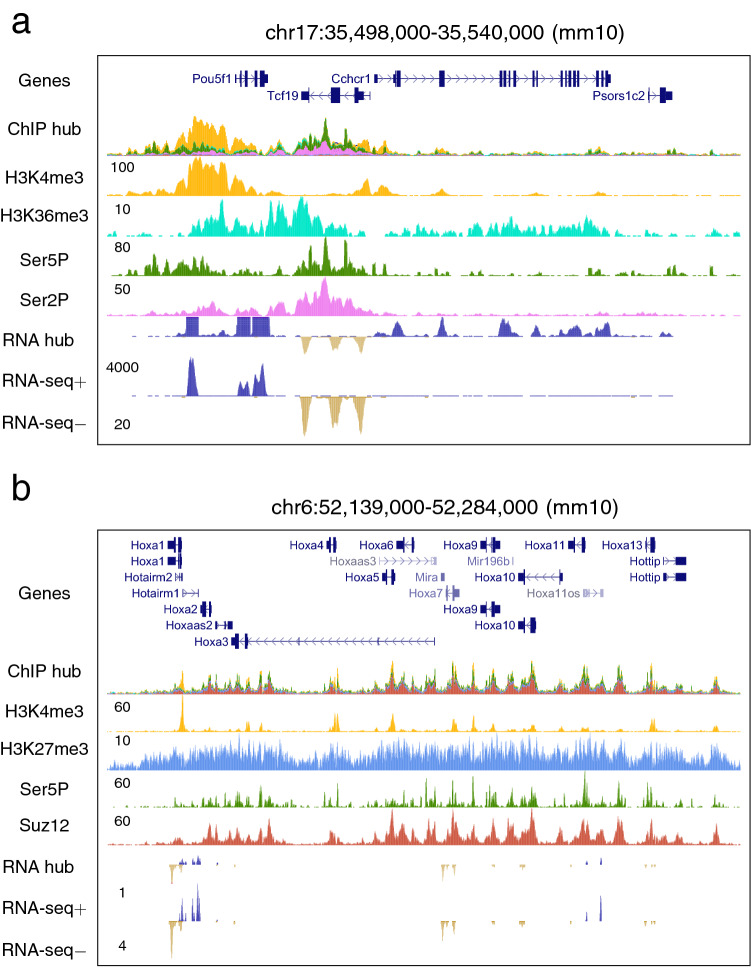
SeqCode ChIP-seq and RNA-seq profiles in mESCs for visualizing in genome browsers. **(a)** Actively transcribed region containing the *Oct4/Pou5f1* pluripotency gene. **(b)** Region repressed for transcription by PcG proteins containing the HoxA complex. Raw data were retrieved from^32,33,38,75^. The SeqCode buildChIPprofile function (window size = 10) was used to generate each custom track from resulting BAM files. The SeqCode combineChIPprofiles function was used to generate the RNA-seq strand-specific profiles. Composite ChIP-seq and RNA-seq supertracks integrate all information from each individual track shown below.

Mapped reads can be piled-up along chromosomes to visually inspect the sequencing profiles in particular regions. The buildChIPprofile function produces genome-wide distributions in BedGraph format from a SAM/BAM file that can be uploaded in genome browsers (Fig. 2 and Supp. Figure 1). Multiple aspects of the profiles are customizable (e.g. name, color, and graphical resolution). SeqCode can also efficiently process paired-end and strand-specific libraries, and spike-in normalization factors can be introduced to weigh the distinct profiles. The distribution of two sequencing experiments can be graphically compared in a single track by subtraction. The combineChIPprofiles function generates genome-wide distributions in BedGraph format from two SAM/BAM files to be visualized in the same manner (see the RNA-seq strand-specific profiles in Fig. 2).

### Averaged occupancy plots to spot the genomic distribution of a transcription factor/histone modification

**Figure 3:**
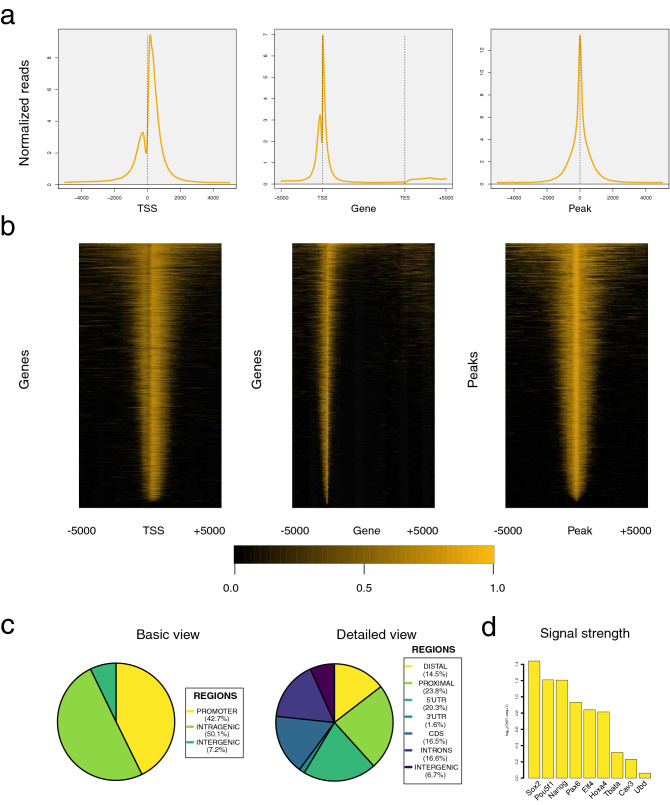
Basic analysis of the H3K4me3 ChIP-seq sample in mESCs using SeqCode. **(a)** From left to right: average distributions around the TSS of H3K4me3 target genes (produceTSSplots), along the gene body of the same genes (produceGENEplots), and around the center of H3K4me3 peaks (producePEAKplots). **(b)** From left to right: heatmap around the TSS of H3K4me3 target genes (produceTSSmaps), along the gene body of the same genes (produceGENEmaps), and around the center of H3K4me3 peaks (producePEAKmaps). **(c)** Basic and detailed genome distribution of H3K4me3 peaks (genomeDistribution). **(d)** ChIP-seq signal levels of selected genes (recoverChIPlevels). Raw data were retrieved from^32^.

Occupancy plots (also known as aggregated plots or meta-plots) show the distribution of functional elements (which are typically reported from a ChIP-seq experiment) around a particular area of selected genes or within genomic intervals, with the aim of uncovering a characteristic pattern. The routines produceTSSplots, produceGENEplots, produceTESplots, and producePEAKplots generate average profiles of large-scale data from BAM files. It is therefore possible to select the viewpoint that is used as a reference to compute the average profile (Fig. 3a), which can be the transcription start site (TSS) of a gene, a normalized meta-gene body (GENE), transcription ending sites (TES), and the center of genomic intervals (PEAK). Moreover, users can configure multiple graphical features of the resulting image (background/foreground colors, flanking area width, etc.). Chromatin accessibility experiments (ATAC-seq) can be processed as well. The occupancy plot of a sequencing experiment can be corrected by subtraction of a second sample with the combineTSSplots function.

### Density heatmaps to display the strength of the signal on each target

Density heatmaps are barcodes that denote the signal strength pattern of a high-throughput experiment from a list of selected genes or a set of genomic intervals. The functions produceTSSmaps, produceGENEmaps, produceTESmaps, and producePEAKmaps generate density heatmaps from a set of target genes or peaks in BED format for a given SAM/BAM file (Fig. 3b). Multiple viewpoints are thus possible: TSS/TES of genes, meta-gene bodies or genomic regions. Customizing the graphical appearance of the final images (e.g. background/foreground colors and resolution) is straightforward in SeqCode (Supp. Figure 2 and our website). A heatmap corresponding to one sequencing experiment can be corrected by a second sample using the combineTSSmaps function.

### Read counts for signal quantification and further dataset comparisons

SeqCode executes the recoverChIPlevels method to determine the maximum, average, and total number of reads of a large-scale experiment within a set of genomic intervals in BED format given in a SAM/BAM file (Fig. 3d). Of note, not only ChIP-seq peaks but also gene annotations converted into this format can be used to evaluate the strength of a sequencing experiment within. Users can choose to normalize the output values with the total number of reads of the experiment or the number of spike-in reads, if available. Resulting distributions can be computed for multiple datasets for further statistical analysis of significance in boxplots or scatter plots.

### Classification of peaks into different genomic features

The genomeDistribution function classifies a list of genomic intervals in BED format (e.g. ChIP-seq peaks) into different features of the genome. Users can produce pie charts and annotation files under distinct degrees of detail (Fig. 3c). Using RefSeq gene transcript annotations, SeqCode classifies each region as promoter (proximal or distal), intergenic, or intragenic (5′ UTR, 3′ UTR, coding sequence [CDS], and introns) (Supp. Figure 3). Peaks overlapping more than one genomic feature are counted as many times as the number of genomic features they contain. On the other hand, superimposed pie charts (spie charts) can highlight the significance of the value distribution elements by using a second distribution as a reference^28^. Thus, when compared to the full-genome distribution of the same features, the results obtained with the genomeDistribution function for a particular sample can be further recycled to generate one spie chart, which depicts the relevance of the frequency of each element in the high-throughput experiment and along the genome (see examples in our website).

### Identification of target genes and peak comparison

ChIP-seq and ATAC-seq peaks are typically associated to the TSS of the nearest gene to further analyze genesets using ontology term enrichment analysis. The matchpeaksgenes routine allows the user to define the conditions of matching a given peak to a certain gene, by defining which regions around the TSS or the gene body (according to RefSeq) can be used in the overlap. Moreover, SeqCode offers a generic version of the same command, called matchpeaks, to compare two lists of genomic intervals in BED format. Overlapping regions and peaks found only in one set are formatted as custom tracks, which can be graphically visualized in genome browsers (see our website for further information).

### Evaluation of the evolutionary conservation of genomic regions

**Figure 4:**
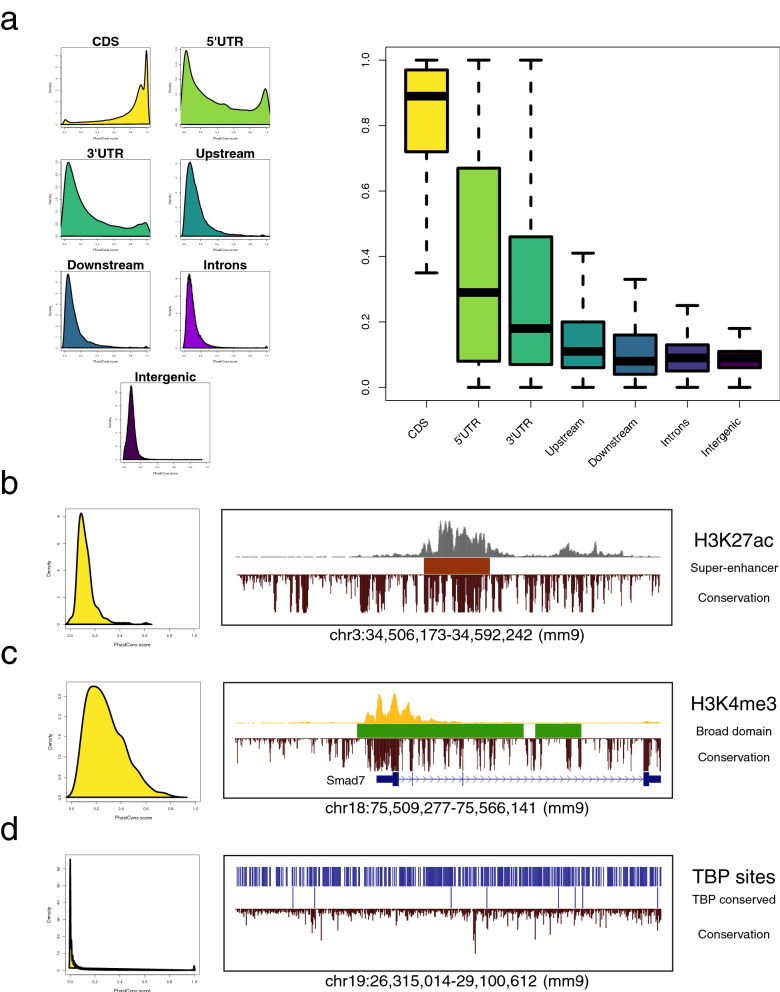
Evolutionary conservation landscape of distinct features of the mouse genome calculated by SeqCode. **(a)** PhastCons average score distribution calculated by the SeqCode scorePhastCons function on distinct gene features: protein-coding regions (CDS), 5′ untranslated regions (5′UTR), 3′ untranslated regions (3′UTR), promoters 1000 bp upstream of the TSS (Upstream), regions 1000 bp downstream the TES (Downstream), intronic regions (Introns), and intergenic regions (Intergenic). RefSeq annotations were used to extract the coordinates of all instances of each feature in the mouse genome (mm9). Left, distribution of the score (from 0 to 1); right, boxplot summarizing differences between features. **(b–d)** PhastCons average score calculated by SeqCode on distinct regulatory features: **(b)** super-enhancers identified as significant concentrations of H3K27ac in mESCs^76^, (red); **(c)** broad domains of H3K4me3 reported in mESCs^77^ (green); and **(d)** computational predictions of TATA boxes using Jaspar^78^, before and after the conservation filtering (TBP and TBP conserved), to highlight the significant drop in the number of ab initio predictions (blue). Left, the global distribution of each class of elements; right, an example of high conservation. The phastCons30way track of mouse was used to score each set of regions with the SeqCode scorePhastCons function. Raw data were retrieved from^32,79^.

Functional sequences of the genome exhibit a strong degree of conservation along evolution. By comparing the sequence of the orthologous region from multiple species, it is possible to score the potential of a genomic interval as containing a conserved regulatory block. The scorePhastCons function takes advantage of the PhastCons score^29^ to rank the members of a peak collection in BED format (note that the PhastCons data files must have been previously downloaded from the UCSC genome browser) (Fig. 4).

### Evaluating the SeqCode soundness

Gene expression is regulated in part by genome organization. For instance, promoters and enhancers associated to transcriptionally active genes must be physically accessible for transcription factors to bind, while silent genes are embedded in genomic regions with a higher level of chromatin compaction^30^. Chromatin remodeling complexes, such as those involving the Polycomb group (PcG) and Trithorax group (TrxG) proteins, modulate nucleosomes by introducing post-translational modifications to the histone tails, which ultimately affect the chromatin conformation^31^. To determine the consistency of SeqCode, we selected the ChIP-seq experiments in mouse embryonic stem cells (mESCs) of two histone modifications: histone H3 lysine 4 trimethylation (H3K4me3) and histone H3 lysine 36 trimethylation (H3K36me3). While H3K4me3 exhibits a characteristic pattern of sharp peaks around TSSs of expressed genes^32^, H3K36me3 is distributed in broad domains entirely covering the bodies of transcriptionally active genes^33^.

We first produced the genome-wide profile of each ChIP-seq experiment using the buildChIPprofile function (Supp. Figure 4) and then generated the average occupancy plot of a single gene comprising both histone modifications using the produceGENEplots function. Custom tracks focused on the same gene, and meta-gene plots coincided well (Supp. Figure 4a). We successfully contrasted the occupancy plot around the TSS of another gene produced by the produceTSSplots function, with the corresponding custom track and its density heatmap generated with the produceTSSmaps function (Supp. Figure 4b). To evaluate the effects of the sequencing depth, we generated genome-wide profiles of both ChIP-seq experiments with the buildChIPprofile function, using different down-sampling sizes. The effect of saturation in both marks is different: one-million reads was sufficient to visualize the H3K4me3 sharp patterning, while additional sequencing depth of eight-million reads was necessary to distinguish the broader domains of H3K36me3 (Supp. Figure 5).

In our second test, we verified that SeqCode offers a good compromise between the quality of the biological results and the amount of available memory in the computer. Users can instruct SeqCode functions to use a particular window resolution in the binning of the genome (option –w), which affects the memory requirements. Although the quality of the final graphical representations was lower (as expected), the results generated with the buildChIPprofile, produceTSSplots and produceTSSmaps programs were consistent in all cases (Supp. Figure 6). Therefore, SeqCode can process a sequencing sample using almost any computer with minimal memory requirements, if necessary (Supp. Figure 7). On the other hand, users working on a powerful workstation can configure this software to obtain the maximum graphical quality.

### Running SeqCode in realistic epigenetic scenarios

Histone marks are thought to be associated with positive and negative transcription events. So-called bivalent domains, first described in mESCs^34^, are simultaneously decorated with a combination of opposing H3K4me3 and histone H3 lysine 27 trimethylation (H3K27me3) marks. Whereas H3K4me3 at the TSS of transcriptionally active genes is a key product of TrxG proteins, H3K27me3 is catalyzed by specific PcG complexes in gene silencing contexts. PcG proteins participate into two different classes of repressive complexes: Polycomb repressive complexes 1 and 2 (PRC1 and PRC2). Each complex comprises a set of core components—such as Ring1b for PRC1, and Suz12 for PRC2—together with other subunits that produce distinct variations of each complex^31^. Bivalent genes and enhancers are key components of the developmental regulatory circuitry, and intensive research in the past years is currently extending the bivalency paradigm to other cellular contexts^35^. Below, we show how to use SeqCode to characterize the epigenomic landscape of different groups of genes in mESCs, and during tumorigenesis, using three practical cases in which bivalency plays a role.

### Case 1. Analysis of the epigenetic signature of the genome

**Figure 5:**
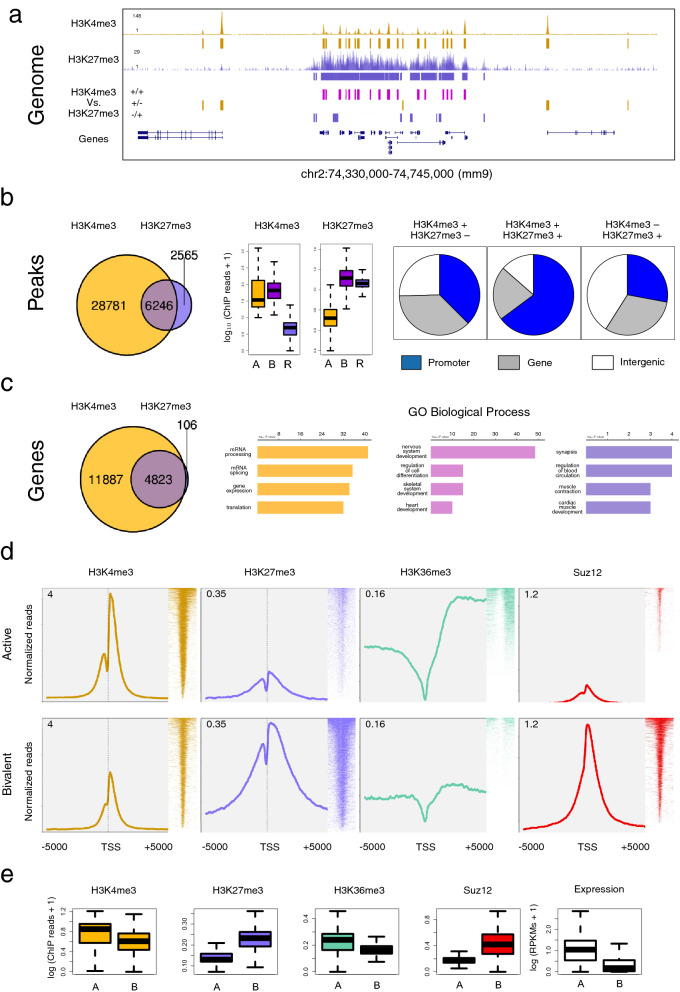
Epigenetic signature of active and bivalent genes from mESCs reconstructed with SeqCode. **(a)** Bivalent region repressed for transcription by PcG proteins containing the HoxD complex. Genome-wide profiles and peaks of H3K4me3 and H3K27me3 are shown along this locus. **(b)** Overlap of H3K4me3 and H3K27me3 peaks produces three classes of sites: active (H3K4me3 + /H3K27me3–; orange), bivalent (H3K4me3 + /H3K27me3 + ; violet), and silent (H3K4me3–/H3K27me3 + ; blue). Determination of signal strength and genomic distribution of each class of peaks. **(c)** Overlap of H3K4me3 and H3K27me3 target genes, showing the functional analysis of active genes (orange), bivalent genes (violet), and silent genes (blue). **(d)** Average distribution and heatmap of the ChIP-seq signal around the TSS of active and bivalent genes for H3K4me3, H3K27me3, H3K36me3, and Suz12. **(e)** ChIP-seq levels of each experiment are shown for active and bivalent genes (left and right boxes in the boxplots, respectively). For comparison, gene expression (in RPKMs) is shown for both gene sets. Raw data of ChIP-seq and RNA-seq experiments were retrieved from^36^. The SeqCode buildChIPprofile function was used to generate each custom track from the resulting BAM files after mapping; the matchpeaks application compared the H3K4me3 and H3K27me3 peaks; the recoverChIPlevels application determined the strength of the ChIP-seq signal at each subset of peaks; the genomeDistribution program calculated the genomic composition of each collection of peaks, according to RefSeq annotations; the matchpeaksgenes routine associated ChIP-seq peaks and target genes; and the produceTSSplots and produceTSSmaps programs generated the average distribution meta-plots and heatmaps of each ChIP-seq sample for signal around the TSS of active and bivalent genes, respectively. GO term enrichment was analyzed with Enrichr^80^. For boxplots in **(b,e)**, ChIP-seq counts were normalized by the total number of mapped reads, and RNA-seq expression values were calculated as RPKMs.

First, we generated the genome-wide profiles of published ChIP-seq experiments of H3K4me3 and H3K27me3^36^ using the buildChIPprofile routine (Fig. 5a). Next, we classified the ChIP-seq peaks available for each histone mark into three distinct classes (H3K4me3 + /H3K27me3–, H3K4me3 + /H3K27me3 + and H3K4me3–/H3K27me3 +) with the matchpeaks function (Fig. 5). Bivalent domains constitute most H3K27me3 peaks. Further ChIP-seq signal quantification of each class of peaks running the recoverChIPlevels program confirmed the composition of each peak class. Bivalency was preferentially found in gene promoters, and in intergenic regions to a minor extent (genomeDistribution pie charts; Fig. 5b). To study in-depth bivalency at the gene level, we ran the matchpeaksgenes application (which here associates a peak to one gene if it is located within 2500 bp from the TSS). Overlap between each set of target genes classified genes into active (H3K4me3 + /H3K27me3–), bivalent (H3K4me3 + /H3K27me3 +), or silent (H3K4me3–/H3K27me3 +). Gene ontology (GO) analyses confirmed that bivalent genes are related to development (Fig. 5c).

In addition to H3K4me3 and H3K27me3, we also characterized active and bivalent genes in terms of H3K36me3 and Suz12 occupancy using the produceTSSplots, produceTSSmaps, and recoverChIPlevels programs (Fig. 5d,e). As expected, H3K4me3 presented a sharp peak centred over the TSS of both classes of genes but which was higher for active genes. For H3K27me3, we observed a canonical broad peak pattern in the TSS of bivalent genes. With respect to H3K36me3, we reproduced the well-known broad domain over the gene body of transcriptionally active genes. Finally, Suz12 was present in the TSS of bivalent genes, which are precisely decorated by H3K27me3. We confirmed the differences at expression levels between both gene groups using RNA-seq data (Fig. 5e).

### Case 2. Characterization of the panel of active and repressive actors in mESCs

We have reconstructed a global picture of this cellular context using ChIP-seq data^32,33,37^ of histone modifications (H3K4me3, H3K36me3, H3K27me3, and ubiquitinated H2A [H2Aub]) and three PcG proteins (Suz12, Jarid2, and Ring1b). PcG proteins have key roles in preventing the transcription of many developmental regulators in mESCs, which would otherwise shift these cells from pluripotency to a more differentiated state^31^. We also integrated ChIP-seq data of two different states of the RNA polymerase II^38^: paused (Ser5-phosphorylated [Ser5P]) and elongating (Ser2-phosphorylated [Ser2P]). Ser5P is a marker of poised promoters, which coincides in most cases with bivalency, while Ser2P is located in gene bodies of transcriptionally active genes^39^. Initially, for each ChIP-seq experiment analyzed here, we retrieved the target peak set from the original publication and used the matchpeaksgenes function to annotate the collection of target genes of each experiment. Next, we have used the produceTSSplots, produceTSSmaps and produceGENEplots functions of SeqCode to study the distribution of each element around the TSS of its target genes.

**Figure 6:**
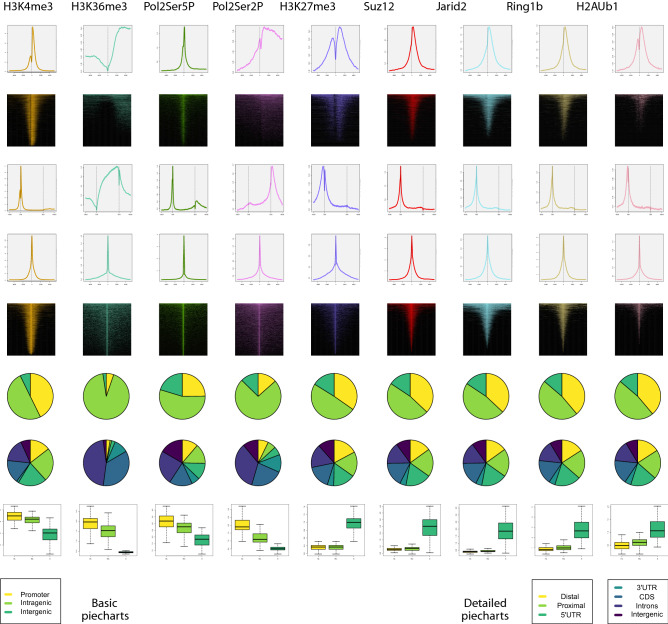
Panel of active and repressive actors in mESCs generated by SeqCode. For each ChIP-seq signal and corresponding set of target genes, the average distribution (produceTSSplots) and heatmap (produceTSSmaps) around the TSS, average distribution along the gene body (produceGENEplots), average distribution (producePEAKplots) and heatmap (producePEAKmaps) around the center of the peaks, genome distribution of each set of peaks (genomeDistribution), and ChIP-seq levels normalized by the total number of reads (recoverChIPlevels) are shown for 100 highly expressed genes (more than 1000 RPKMs; red), 100 moderately expressed genes (100–500 RPKMs; yellow) and 100 silenced genes (0–1 RPKMs; blue). Raw data were retrieved from^32,33,37,38^. Target genes of each ChIP-seq were identified with the matchpeaksgenes routine.

Analysis of meta-gene plots (Fig. 6) indicated that the highest ChIP-seq signal was found around the TSS, with the exception of H3K36me3 and Ser2P, which presented a broad pattern covering gene bodies. In line with this, similar differences were observed when analyzing the genome distribution of peaks of each experiment (Fig. 6): while H3K36me3 and Ser2P pie charts generated using the genomeDistribution command were enriched in intragenic categories, the remaining elements tended to be located around the TSS of genes. Intensity of ChIP-seq signals around the center of peaks was reported using the producePEAKplots and producePEAKmaps commands. Finally, we performed a random sampling of RefSeq genes, in three groups of 100 genes each according to RNA-seq expression, of highly expressed, moderately expressed, or silent (Fig. 6, lower panel). By calculating the ChIP-seq normalized counts of each experiment along the three gene classes with the recoverChIPlevels function, a clear pattern emerged: gene expression positively correlated with H3K4me3, H3K36me3, Ser5P, and Ser2P, while it negatively correlated with H3K27me3, Suz12, Jarid2, Ring1b, and H2Aub (Fig. 6). We successfully used the same approach to study the epigenetic landscape of the wing imaginal disc of the fruit fly, using distinct histone marks combined with transcription factor binding (Supp. Figure 8). Both examples illustrate how SeqCode can be executed to build comparative panels of a high number of sequencing samples, which can then be used to obtain novel knowledge for further analysis.

### Case 3. Comparative analysis after genetic perturbation of epigenetic actors

Although ChIP-seq experiments are semiquantitative approaches to evaluate the presence of a certain histone modification or protein on a genomic region, they can be relevant to compare the outcome for the same antibody in two different conditions. Genetic deletion of a gene can be used as validation for this scenario. Thus, the ChIP-seq pattern of a target protein in wild-type condition (WT) should be totally (or at least partially) erased when performing ChIP-seq analysis using the same antibody in knockout (KO) or knockdown (KD) cells. Moreover, once the effect of the perturbation has been performed, researchers often evaluate its effect, for example, over putative alterations of the ChIP-seq pattern of a second biological target.

**Figure 7:**
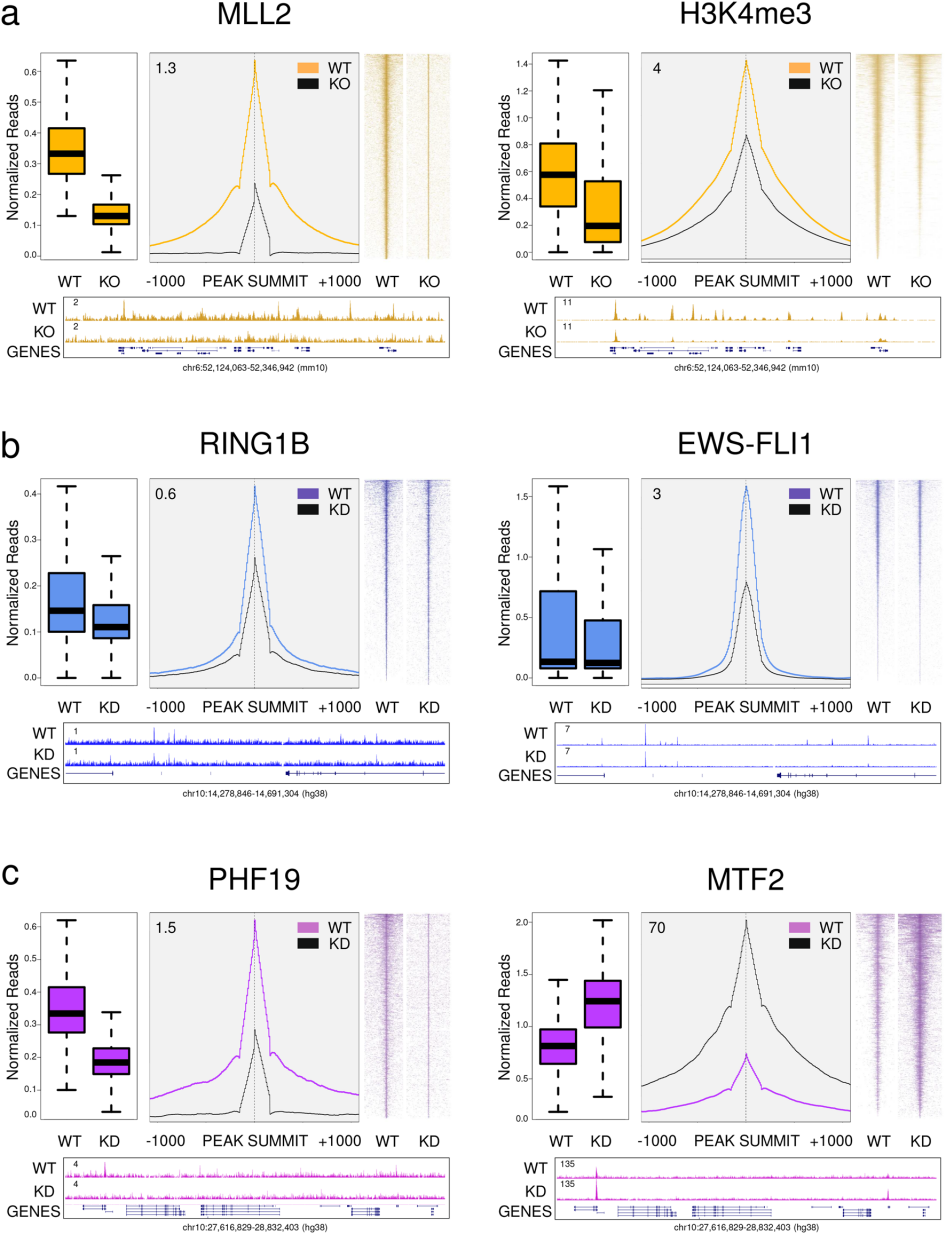
Evaluation of ChIP-seq experiments after genetic perturbation with SeqCode. **(a)** Study of the efficiency of MLL2 knockout (KO) in mESCs (left) and its effect over the occupancy pattern of H3K4me3 (right). **(b)** Study of the efficiency of RING1B knockdown (KD) in the Ewing sarcoma A673 cell line (left) and its effect over the binding pattern of EWS-FLI1 oncogenic protein (right). **(c)** Study of the efficiency of PHF19 KD in the prostatic cancer DU145 cell line (left) and its effect over the pattern of MTF2 (right). Peaks called in the wild-type (WT) condition of the factor being analyzed on each case were used as a reference in both conditions. For every pair of WT-KO/KD experiments, ChIP-seq normalized levels (recoverChIPlevels), and the average distribution (producePEAKplots) and heatmap (producePEAKmaps) around the peaks are shown. Below, genome-wide screenshots of the WT and KO/KD profiles on a locus containing peaks of this class were generated with the SeqCode buildChIPprofile function. Boxplots of ChIP-seq values are shown in log scale. All samples were normalized by the total number of reads of each experiment, except MTF2 ChIP-seq samples that were normalized by the total number of spike-in fruit-fly reads. Raw data were retrieved from^40–42^.

To illustrate both concepts, we have revisited three scenarios gathered from the literature. In first place, we depicted the intensity of ChIP-seq of MLL2^40^, a TrxG protein required for H3K4me3 deposition associated to bivalent promoters in mESCs, under two conditions (WT and MLL2 KO) using the recoverChIPlevels, producePEAKplots, producePEAKmaps, and buildChIPprofile functions. In all the graphical representations, a substantial drop in the ChIP-seq signal of MLL2 upon KO of MLL2 is reported, as expected (Fig. 7a, left). Importantly, this event is accompanied by the corresponding decrease in the H3K4me3 product (Fig. 7a, right).

Secondly, we explored how the role of PcG components is crucial also in the context of cancer. In fact, when scrutinizing with SeqCode the ChIP-seq profiles of RING1B (a PRC1 subunit) for Ewing sarcoma (EWS)^41^, we confirmed (i) the good performance of the RING1B KD, and (ii) a striking decrease in the binding of the EWSR1-FLI1 fusion protein, which is responsible for the EWS disease, upon KD of RING1B (Fig. 7b). Finally, in prostate cancer, we employed a similar strategy to evaluate the effects of the KD of PHF19, a PRC2 subunit, using SeqCode. In this case, we observed an increase of MTF2 (also a PRC2 subunit) upon PHF19 KD (Fig. 7c), suggesting the existence of a compensatory mechanism^42^.

### Offering SeqCode functions through a front-end web site

**Figure 8:**
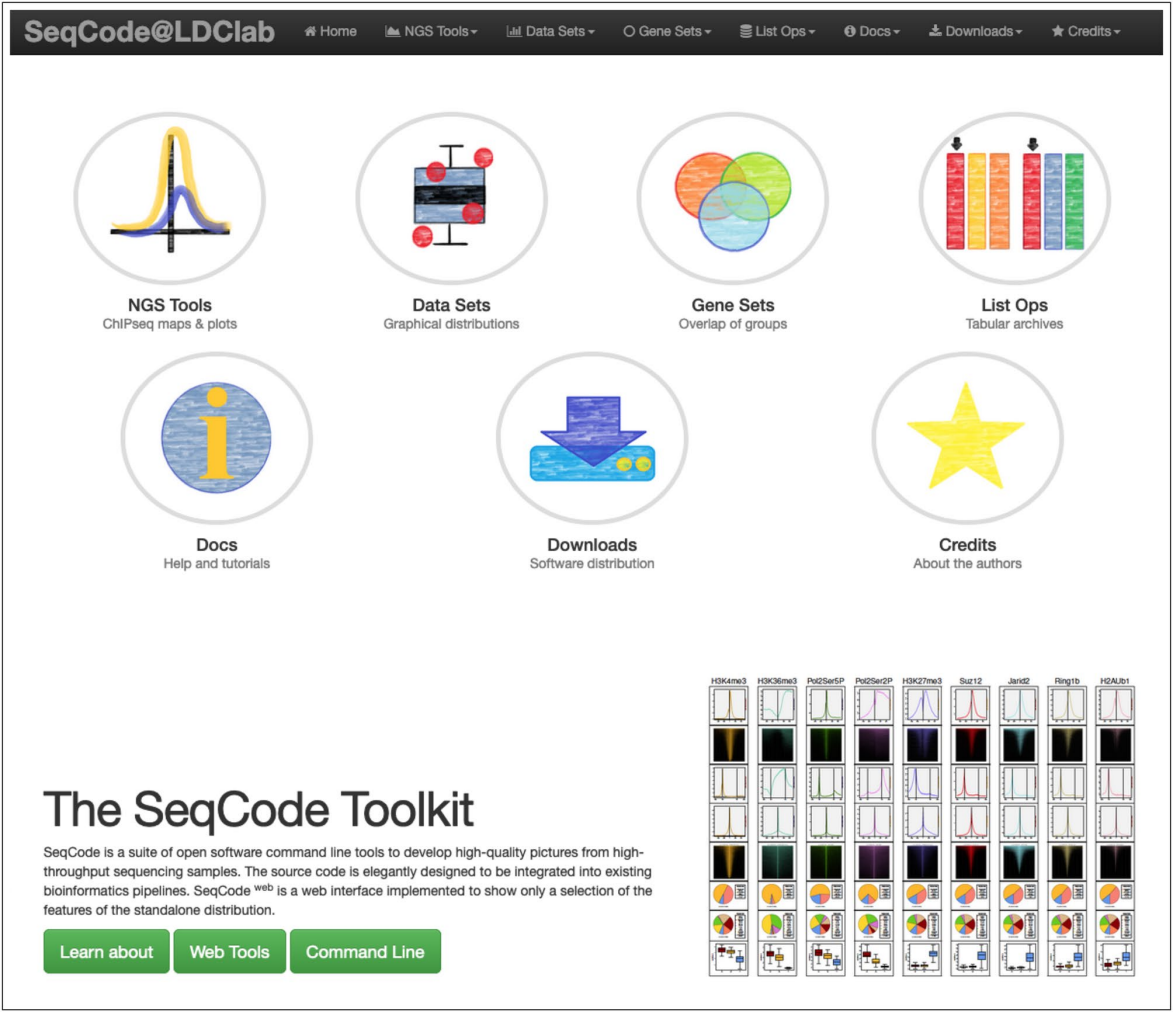
Homepage of the SeqCode website. The main menu of the web server is divided into four categories: NGS Tools (sequencing plots), Datasets (distribution plots), Gene Sets (intersection plots) and List Ops (operations with lists of identifiers). Additional help documentation and a section with downloading links are also provided here.

This software can be potentially integrated as an external component of any bioinformatics pipeline. For that purpose, we have designed a user-friendly web interface to grant access to most functions of our software (http://ldicrocelab.crg.eu/). Our website homepage is structured into four classes of services (Fig. 8):

(1) NGS tools (Supp. Figure 9): sequencing analysis tools (ChIP-seq peak annotation and gene association, signal quantification, aggregated plots, and density heatmaps), for a selection of published ChIP-seq experiments;

(2) Datasets (Supp. Figure 10): functions to graphically represent user data distributions also in form of correlations or clusters (e.g. boxplots, PCA plots, heatmaps and scatter plots);

(3) Gene sets (Supp. Figure 11): plots to compare the overlap between members of distinct user datasets (e.g. Venn diagrams, alluvial charts and UpSet plots^27^);

(4) List Ops: operations between lists of values provided as plain text files. Each line of information contains a record, and each column contains the value for a particular feature (e.g. joining two files, filtering in one file of specific records, etc.).

In most cases, users will find options for appropriate labelling and customization of most graphical parameters of the corresponding SeqCode web service. Each application generates the images in PNG and PDF formats and provides links to the source files and R scripts. Users will find abundant on-line documentation about each service and a comprehensive set of tutorials. Moreover, we have integrated a complete manual of the command-line version of SeqCode, furnished with multiple examples of analysis, into our website.

### Comparison with similar tools

**Figure 9:**
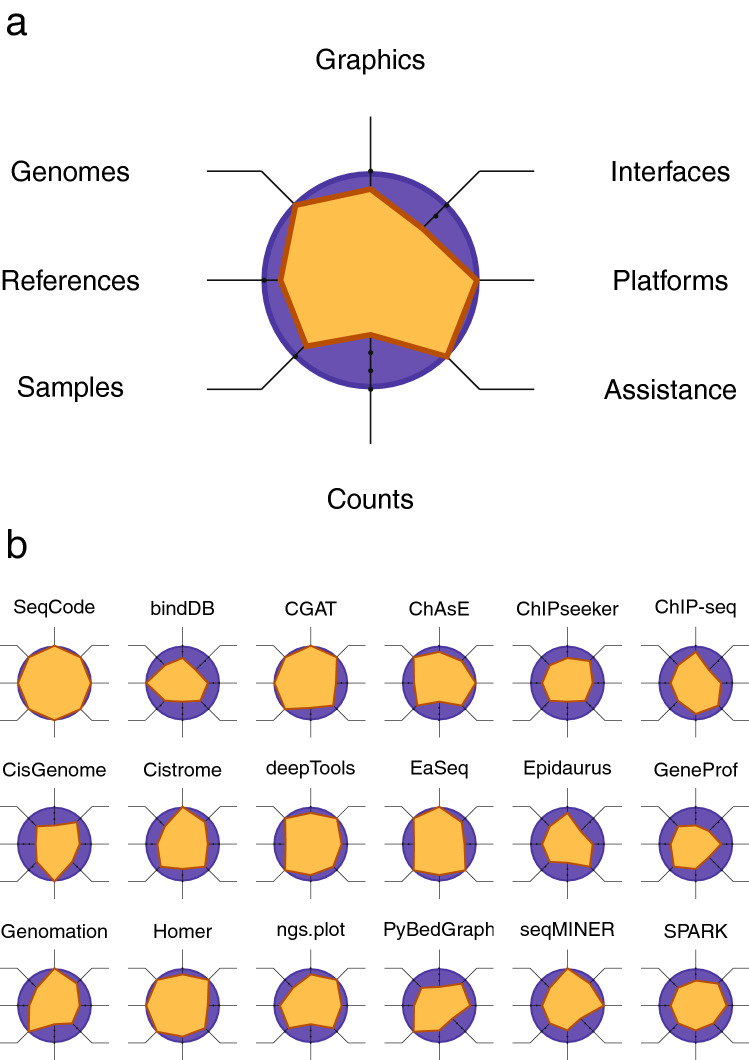
Evaluation of tools for the visualization of sequencing data. **(a)** Radar chart displaying the eight attributes that have been used to describe the characteristics of the existing applications. **(b)** Radar charts of each selected bioinformatics software for graphical summarizing sequencing data (see Table 3 for the values that are graphically displayed for each program here).

Assessing the available software for graphical annotation of large-scale sequencing information is complex due to the heterogeneity of the applications and their potential audiences^43–45^. Thus, certain tools such as SAMtools^18^, BEDTools^19^, and FAST^46^—which are designed to manage NGS information at a low level—will be excluded from this comparative study. On the other hand, instead of GALAXY^17^, the major hub of web interfaces towards other NGS applications, we opted for evaluating Cistrome^44^, a GALAXY-based server, and deepTools^47^, a visualization resource also available inside GALAXY. Here, we evaluated 18 different visualization tools, which to our knowledge constitutes the most comprehensive selection to date and provides a faithful portrait of the current state of art in this field (Table 2). We propose to focus on eight elementary features that characterize every program (Fig. 9a): (i) the inventory of genomes that can be analyzed, (ii) the class of genomic element that can be interrogated, (iii) the type of sequencing data to be processed, (iv) the family of graphical representations that can be generated, (v) the available options for determining signal strength of samples, (vi) the flexibility of the working interface for analysis, (vii) the computational requirements to be satisfied to run the software, and (viii) the state of completion of the final documentation provided to the users. The final scores of each program according to these attributes are summarized in Fig. 9b and Table 3, and examples of output from several tools reproducing our analysis of active genes, bivalent genes, or repressed genes in mESCs (see Fig. 5) are shown in Supp. Figure 12–14.

**Table 2:** List of bioinformatics tools for graphical analysis of sequencing information.

Software	References
SeqCode	This article
BindDB	Livyatan et al.^81^
CGAT	Sims et al.^82^
ChAsE	Younesy et al.^83^
ChIPseeker	Yu et al.^84^
ChIP-seq tools	Ambrosini et al.^85^
CisGenome	Ji et al.^86^
Cistrome	Liu et al.^44^, Zheng et al.^87^
deepTools	Ramirez et al.^47^, Ramirez et al.^88^
EaSeq	Lerdrup et al.^43^
Epidaurus	Wang et al.^89^
GeneProf	Halbritter et al.^90^
Genomation	Akalin et al.^91^
HOMER	Heinz et al.^92^
ngs.plot	Shen et al.^45^
PyBedGraph	Zhang et al.^93^
seqMINER	Ye et al.^94^
Spark	Nielsen et al.^95^

**Table 3:** Characteristic features of SeqCode and other similar resources.

Feature	SeqCode	BindDB	CGAT	ChAsE	ChIPseeker	ChIP-seq	CisGenome	Cistrome	deepTools	EaSeq	Epidaurus	GeneProf	genomation	HOMER	ngs.plot	PyBedGraph	seqMINER	Spark
**Genomes**																		
1. Selection	X	X	X	X	X	X	X	X	X	X	X	X	X	X	X		X	X
2. All genomes	X		X	X					X	X				X				
3. All assemblies	X		X	X					X	X				X		X		
**Reference**																		
1. Gene	X	X	X					X			X	X		X				
2. Enhancer	X	X	X											X	X			
3. Genomic	X	X	X	X	X	X			X	X			X	X	X	X	X	X
**Samples**																		
1. ChIP-seq	X	X	X	X	X	X	X	X	X	X	X	X	X	X	X	X	X	X
2. RNA-seq	X		X						X	X		X	X	X	X	X		
3. ATAC-seq	X		X	X				X	X	X			X	X		X		
**Graphics**																		
1. Aggregated	X		X	X		X		X	X	X	X		X	X	X		X	
2. Heatmaps	X	X	X	X				X	X	X	X		X		X		X	X
3. Distributions	X		X		X	X		X		X			X	X			X	
**Counts**																		
1. Custom tracks	X		X			X	X		X	X		X		X				X
2. Signal strength	X						X		X	X				X		X	X	
3. Conservation	X					X	X	X										
**Interface**																		
1. Local	X		X	X	X		X		`X	X			X	X	X	X	X	X
2. Flexible	X		X					X	X					X	X			
3. Dynamic	X		X	X	X		X	X	X	X			X	X	X	X	X	X
**Platform**																		
1. Executable	X			X						X							X	X
2. Parametric	X			X					X						X	X	X	
3. Portable	X	X	X	X	X	X	X	X	X		X	X	X	X	X	X	X	X
**Documents**																		
1. Web site	X	X	X	X	X	X	X	X	X	X	X		X	X	X			X
2. Tutorials	X		X	X		X		X	X	X	X			X	X			
3. Self-docs	X									X								

### (i) Which inventory of genomes can be analyzed?

Most programs work only for a small subset of species, mostly human and mouse (GeneProf, BindDB) and perhaps also other model organisms, such as the fruit fly (ChIP-Seq, CisGenome). Certain programs provide access only to a subset of genome assemblies for these species (Cistrome, ngs.plot). To circumvent these issues, EaSeq provides a connection to link available annotations in the UCSC genome browser to elements into the analysis pipeline. Other tools allow externally annotations for other genomes to be introduced using GTF files that must be previously customized by the user (CGAT, HOMER). In contrast to these, SeqCode adopts the standard annotation files provided by the RefSeq consortium for almost any genome. This strategy permits users to easily work with different releases of the same genome by simply switching to the required RefSeq gene annotation file.

### (ii) Which class of genomic information can be interrogated?

Most resources perform actions exclusively focusing on a single class of element as the principal viewpoint. Thus, users can interrogate sequencing data using gene names (GeneProf and Cistrome) or genomic intervals defining ChIP-seq peaks (ChIP-Seq, ChIPseeker, deepTools, EaSeq, genomation, seqMINER and Spark). However, depending on the context, dual access through both classes of reference points (genes and peaks) is frequently convenient throughout most bioinformatics analysis, which usually combine ChIP-seq binding data with RNA-seq expression data. Therefore, although it is not yet a universal feature, SeqCode and other tools such as BindDB, CGAT and ngs.plot allow the user to characterize sets of genomic intervals (e.g. enhancers) together with gene regions based on their functions.

### (iii) Which class of sequencing experiments can be processed?

Many applications are uniquely designed to analyze ChIP-seq data. However, other NGS methods, including RNA-seq, ATAC-seq, and MeDIP-seq, are widely accessible to the scientific community. We found a variety of combinations in the type of experiment that can be analyzed: GeneProf and ngs.plot, ChIP-seq and RNA-seq; Cistrome, ChIP-seq and ATAC-seq; and ChAsE, ChIP-seq and MeDIP-seq. There is an emerging group of general-purpose programs that can manage all class of NGS information: SeqCode (presented here), CGAT, deepTools, EaSeq, Genomation, and HOMER. To our knowledge, SeqCode is the only software in which all the routines can be applied over the reads aligned in a BAM file irrespectively of the class of sequencing experiment (e.g. paired-end or single-end). This allows users to easily add spike-in corrections to the data normalization of every class of experiment.

### (iv) What families of graphical representations can be generated?

The vast majority of applications (CGAT, ChAsE, Cistrome, Epidaurus, genomation, HOMER, and seqMINER) generate at least basic occupancy aggregated plots or heatmaps of ChIP-seq samples. However, the viewpoint is usually anchored to a single point (e.g. TSS or peak center), and representations along meta-gene bodies and genomic intervals are rarely available. Notably, certain tools (SeqCode, deepTools, EaSeq, and ngs.plot) are also able to generate heatmaps along gene bodies mimicking meta-gene aggregated plots. Finally, while annotation of genomic intervals into different features of the genes is available in most approaches, SeqCode provides highly customized graphics following multiple rules of genomic association, with different degrees of detail.

### (v) What methods are available to evaluate the experimental signal strength?

Functions for read counting are extremely useful for bioinformaticians. Visualization of genome-wide signal profiles reveals at first sight the outcome of fresh NGS experiments. Determination of the amount of signal of an experiment within particular intervals of genomic regions can be used to calculate how well two or more sets of peaks, genes, or experiments are correlated. Only a handful of tools provide options for quantification (SeqCode, deepTools, EaSeq, and HOMER), while SeqCode is the only one to provide options to normalize the results (e.g. profiles and values) and to allow for spike-in correction of all routines. SeqCode (along with ChIP-seq, Cistrome and HOMER) also supports phylogenetic footprinting assessment of evolutionary information on sequencing data.

### (vi) How efficient is the access to the information through the user interface?

User-friendly websites (BindDB, ChIP-seq, Cistrome and Epidaurus) typically work without installing additional software. This means that elevated transfer time of samples through the Internet is a limiting factor, reducing the analysis over a static repository. In contrast, while local-based tools (such as SeqCode, CGAT, ChAsE, EaSeq, HOMER, ngs.plot and seqMINER) require initial setup, they are able to dynamically process user sequencing experiments with a much shorter time of response. Graphical interfaces are accessible, but command-line environments are more flexible for customizing each function and automatize batch processing. Hybrid software combining web servers and local interfaces is less frequent (ChIP-seq, Cistrome, and deepTools). We have designed the web interface of SeqCode with the aim of providing a graphical interface to command-line functions but also to build a solid educational platform to learn the basic analytical procedures of NGS data.

### (vii) Which are the computational requirements to run this software?

Web resources (BindDB, ChIP-seq, and Cistrome) appear to facilitate users to ignore processing power and memory capacity, but their use is limited in realistic scenarios. Standalone programs for professional sequencing analysis, although more efficient, exhibit distinct throughput, depending on their internal implementation. As a rule of thumb^48^, executable binaries (SeqCode and EaSeq) are less time-consuming than Java-based systems (CisGenome, seqMINER, ChAsE), scripting approaches (CGAT, deepTools, HOMER), and libraries developed for R (genomation, ngs.plot, ChIPseeker). Strikingly, only a few methods pay attention to customizing the usage of memory (SeqCode, ChAsE, deepTools, and seqMINER), allowing the analysis to be performed in every computational configuration without affecting the biological conclusions of the experiments. Virtualization of these services and portability are key properties to allowing users to launch and test the software over any platform. Of note, only SeqCode and deepTools offer this service, through virtual machines and docker applications.

### (viii) How easy is accessing information and getting assistance for users?

Inexpert users could be interested in learning the basic use for each function, while more experienced users might wish to generate more complex analyses. However, the majority of the available programs provide minimal documentation addressing these issues (such as README files, manual of functions, working case tutorials, videos). In addition to SeqCode, we only found two other programs (deepTools and EaSeq) that provide comprehensive descriptions of programs and examples. SeqCode and EaSeq also integrate textual descriptions and additional pieces of information included with the final PDF files, which can be very useful for interpreting the final results.

## Discussion

High-throughput experiments are fundamental to current research in molecular biology in the data-driven hypothesis paradigm. However, certain aspects of the analysis of sequencing still creates a barrier for the democratization of access to such technologies, to all level of users. For instance, computational performance requirements and storage capacities that are necessary to run most applications are still being improved^49–51^. Standardization of graphical data representation is also important^52^: most bioinformatics pipelines mainly focus on the mapping and counting stages, with moderate interest for visualization of the resulting information. It is very difficult for the bioinformatician to choose the appropriate software once the primary analysis of the sequencing experiment is done, as existing methods to generate graphical representations of the results do not completely solve the problem. Limited reproducibility among methods and the complex technical set-up of such tools further compromise their use. Indeed, when trying to recreate the bivalency analysis (Fig. 5) using other programs, we noted that certain functions are available in one program but not in another, and detected that most graphical representations of the same data are similar but not identical (Supp. Figure 12–14). Of note, recent efforts on formally defining a uniform framework for genomic data visualization promise to revolutionize this area of research. Thus, Gehlenborg and colleagues^53^ classified genomic information into three distinct taxonomies: data (sparse/contiguous nature and interconnections), visualization (coordinate system, tracks, and views), and tasks (searches and queries). Interestingly, such principles of design have been successfully applied to build interactive and scalable genomics visualizations^54^.

We introduce SeqCode here to bridge this gap in visualizing processed data in a thorough manner. Our main purpose has been to build a software that performs most graphical operations on sequencing data with a quick response time and that is functional in most computational environments. One could argue that, biased for the type of representations constructed here (e.g. meta-plots, heatmaps, boxplots, etc.), SeqCode is more appropriate for analysis of local genomic information, which is produced in form of static high-resolution plots for publication. However, we consider that through the combination of genome-wide profiles and lists of biological features ranked by signal strength and gene associations generated by SeqCode, users will efficiently perform global analytical tasks on their NGS datasets too. Ultimately, the selection of a particular tool to generate a certain data visualization mostly depends on the biological question to address (Supp. Figure 15). Although several commands can be suitable to give response to a particular need, the pros and cons of each visualization, usually due the nature of the data graphically represented, must be rigorously considered. Thus, while genome-wide profiles displayed along chromosomes are a simple way to visually inspect experiments, systematic approaches such as meta-plots and heatmaps provide a more effective way to identify characteristic trends at a glance. On the other hand, average values reported in meta-plots can be seriously affected by the existence of genomic loci with an aberrant number of sequencing events^55^. Similarly, although heatmaps graphically represent the whole map of signal values and are extremely useful to highlight clusters of elements presenting distinct patterns, boxplots are necessary to study the distribution of signal strength along the full collection of elements (e.g. genes, enhancers). Pie charts, which are typically used to represent the abundance of a particular feature for different genomic regions, are recently under discussion and often substituted by bar plots^56^. We recommend to integrate each available visualization that might address a biological question to generate a consistent interpretation of the data.

The unit of information in SeqCode is the RefSeq annotation file, which is provided by most genome browsers; therefore, our applications are usable over every existing genome assembly. Notably, and in contrast to complex graphical and command-line interfaces based in configuration files, SeqCode provides simple but effective options to customize the final results. How to choose the appropriate palette of colors to generate the final graphical representation becomes a fundamental question^57^. We have also implemented options in our software to address the latest findings on analysis of NGS data. For instance, although the relationship between ChIP-seq signal strength and the functionality of a biological feature is still under investigation, significant progress has been based on performing quantitative comparisons with spike-in methods^58,59^. Thus, we allow users of SeqCode to correct the normalization of sequencing samples by introducing the spike-in factor in our applications. This option has recently proven to be useful to successfully evaluate different strategies of data normalization^60^. Indeed, proper graphical comparison of multiple sets of NGS samples corresponding to distinct biological scenarios (Fig. 7), and evaluation of consistency in the replicates of the same experiment coupled with quality control metrics^61^ are increasingly becoming fundamental analytical tasks. Thus, we envisage that future developments in SeqCode will integrate tools to systematically automatize such set of comparative procedures.

SeqCode is entirely written in standard C, which makes this tool the best choice for fast visualization of the results, as compared to other programs designed with scripting languages that require more time to process huge volumes of information. Despite our efforts to cover the majority of graphical summary classes, we cannot discard, though, that we have omitted other graphical visualization modes or data formats. Generation of genome-wide profiles in bigwig format^62,63^, for instance, should be considered in future versions. However, we believe that the open architectural design of SeqCode favors the easy incorporation of new components while preserving its common interface and performance. In fact, as the SeqCode source code is open and freely distributed through GitHub, other developers can introduce improvements and new functions in a simple manner to adapt it to specific issues. We have comprehensively scrutinized the portability of our software under distinct UNIX-like platforms (i.e. macOS and Linux). However, to approach users with different degrees of expertise and prevent potential issues due to different compiler versions in certain platforms, we have distributed our software in other running environments, such as Docker containers or Linux virtual machines loaded with a pre-installation of SeqCode inside. We have also designed a web interface to cover most SeqCode functions that incorporates additional useful tools for data plotting. We would like to stress that the interoperability provided through both SeqCode standalone and web platforms, will fulfil most needs of our potential audience. Therefore, SeqCode can be easily adopted as another component of a comprehensive computational pipeline for analyzing sequencing data, such as ChIP-seq, RNA-seq, or ATAC-seq, once quality control, read mapping, and counting have been performed. In sum, after this thorough evaluation of existing tools, we consider SeqCode to be the most interesting option for analysts of NGS data with different backgrounds. We have already successfully used SeqCode in multiple research projects^36,40,64–70^.

Last but not least, education of future bioinformaticians is increasingly becoming a hot topic in the field^71–73^, and we would like to underscore that we specifically designed an open web and command-line platform that can be integrated into any educational program focused on the high-throughput analysis of genomic data. Feedback on the use of our software by teachers for such a topic is strongly welcome.

## Conclusions

To sum up, we consider that SeqCode will become a fundamental tool to deal with NGS experiments that require a fast and complete analysis and will be the most valuable option for a wide range of users.

## Methods

The full source code distribution of SeqCode can be downloaded from GitHub (https://github.com/eblancoga/seqcode). Our software during the execution initially loads the information from the chromosome size and RefSeq transcript files provided by the user to construct an image of each chromosome in memory. Chromosomes are divided into series of consecutive fragments of the same size, which are termed bins. The size of the bin is configurable by the user. This segmentation is therefore useful during the calculations of values for all the graphical representations generated by SeqCode. Our programs internally integrate the SamTools and HTS C libraries^18^ in order to efficiently read BAM and SAM files. The SeqCode web site (http://ldicrocelab.crg.eu/) is implemented in PHP and the output of graphical services is provided in PNG and PDF formats. Basic R core packages are utilized to produce the resulting plots of all SeqCode functions.

Raw data of the examples introduced throughout the article (Table 4) were retrieved from the NCBI GEO repository^74^. Bowtie^7^ was used to map the reads over the genome of mouse (mm9 and mm10), human (hg38), and the fruit fly (dm3). Unaligned reads must be excluded from the resulting BAM files that will be processed by SeqCode commands. To ensure a fair comparison among samples, SeqCode performed the normalization of values calculated within any finite element (e.g. regions, bins, etc.) for a given sequencing experiment by sequencing depth (i.e. total number of reads in the BAM file). When spike-in material was included into the ChIP-seq experiment (e.g. MTF2 samples in Fig. 7), the number of reads mapped over the spike-in genome was instead used to normalize the data. MACS^12^ was used to perform the peak calling of the ChIP-seq experiments. Custom tracks generated with the buildChIPprofile function of SeqCode follow the 0-start, half-open coordinate counting system. Screenshots were captured with the UCSC genome browser^14^. To speed up the generation of plots with produceTSSplots, produceGENEplots, produceTSSmaps, and computeChIPlevels web services for the genes provided by the user, visualization data of the whole catalog of genes were previously generated on each predefined ChIP-seq experiment with the option –g of such commands (Supplementary information).

**Table 4:** List of GEO accession codes of the raw data analyzed in this work.

GEO accession	Sample	Cell/Tissue	References
SRX367147	H3K4me3	mESCs	Tee et al.^32^
SRX336228	Jarid2		
GSM1019769	H3K36me3		Ballare et al.^33^
GSM1019772	H3K27me3		
GSM1019771	Suz12		
GSM850467	Ser5P		Brookes et al.^38^
GSM850470	Ser2P		
GSM850471	H2Aub1		
GSM1562339	RNA-seq		Jacinto et al.^75^
GSM1526287	H3K27ac		Ji et al.^79^
GSM2098958	H3K4me3		Beringer et al.^36^
GSM2098952	H3K27me3		
GSM1041372	Ring1b		Morey et al.^37^
GSM2645501	MLL2 WT		Mas et al.^40^
GSM2645502	MLL2 KO		
GSM2645495	H3K4me3 WT		
GSM2645496	H3K4me3 KO		
GSM4616798	RING1B WT	A673	Sánchez et al.^41^
GSM4616801	RING1B KD		
GSM4616799	EWSR1-FLI1 WT		
GSM4616802	EWSR1-FLI1 KD		
GSM4023634	PHF19 WT	DU145	Jain et al.^42^
GSM4023635	PHF19 KD		
GSM4023643	MTF2 WT		
GSM4023644	MTF2 KD		
GSM593408	H3K4me3	Wing imaginal disc (fly)	Perez-Lluch et al.^96^
GSM593409	H3K27me3		
GSM593410	H3K36me3		
GSM593412	Ser5P		
GSM593411	Ser2P		
GSM1182471	H3K4me1		Perez-Lluch et al.^97^
GSM1182472	H3K27ac		
GSM1363590	H3K9ac		
GSM1060715	RNA-seq		
GSM1005586	Cabut		Ruiz-Romero et al.^98^

## ﻿Supplementary Information

Supplementary Figure S1.

Supplementary Figure S2.

Supplementary Figure S3.

Supplementary Figure S4.

Supplementary Figure S5.

Supplementary Figure S2.

Supplementary Figure S7.

Supplementary Figure S8.

Supplementary Figure S9.

Supplementary Figure S10.

Supplementary Figure S11.

Supplementary Figure S12.

Supplementary Figure S13.

Supplementary Figure S14.

Supplementary Figure S15.

Supplementary Legends.

## Data Availability

The SeqCode website is hosted at http://ldicrocelab.crg.eu. The SeqCode software is freely available under the GNU General Public License v3.0. Source code has been deposited at the GitHub repository (https://github.com/eblancoga/seqcode). To improve the portability of the software, we also provide Oracle VM virtualbox™ appliances and Docker containers built on Linux Ubuntu MATE at our web site (http://ldicrocelab.crg.eu/06_Downloads/). The datasets analyzed during the current study are available in the GEO repository (see Table 4).
