# Supplementary material for: Productive visualization of high-throughput sequencing data using the SeqCode open portable platform
Source: Sci Rep. 2021 Oct 1;11:19545. doi: 10.1038/s41598-021-98889-7 (PMC8486768; doi:10.1038/s41598-021-98889-7)
Supplement: Supplementary file 1 — Supplementary Figure S1. [file 41598_2021_98889_MOESM1_ESM.pdf]

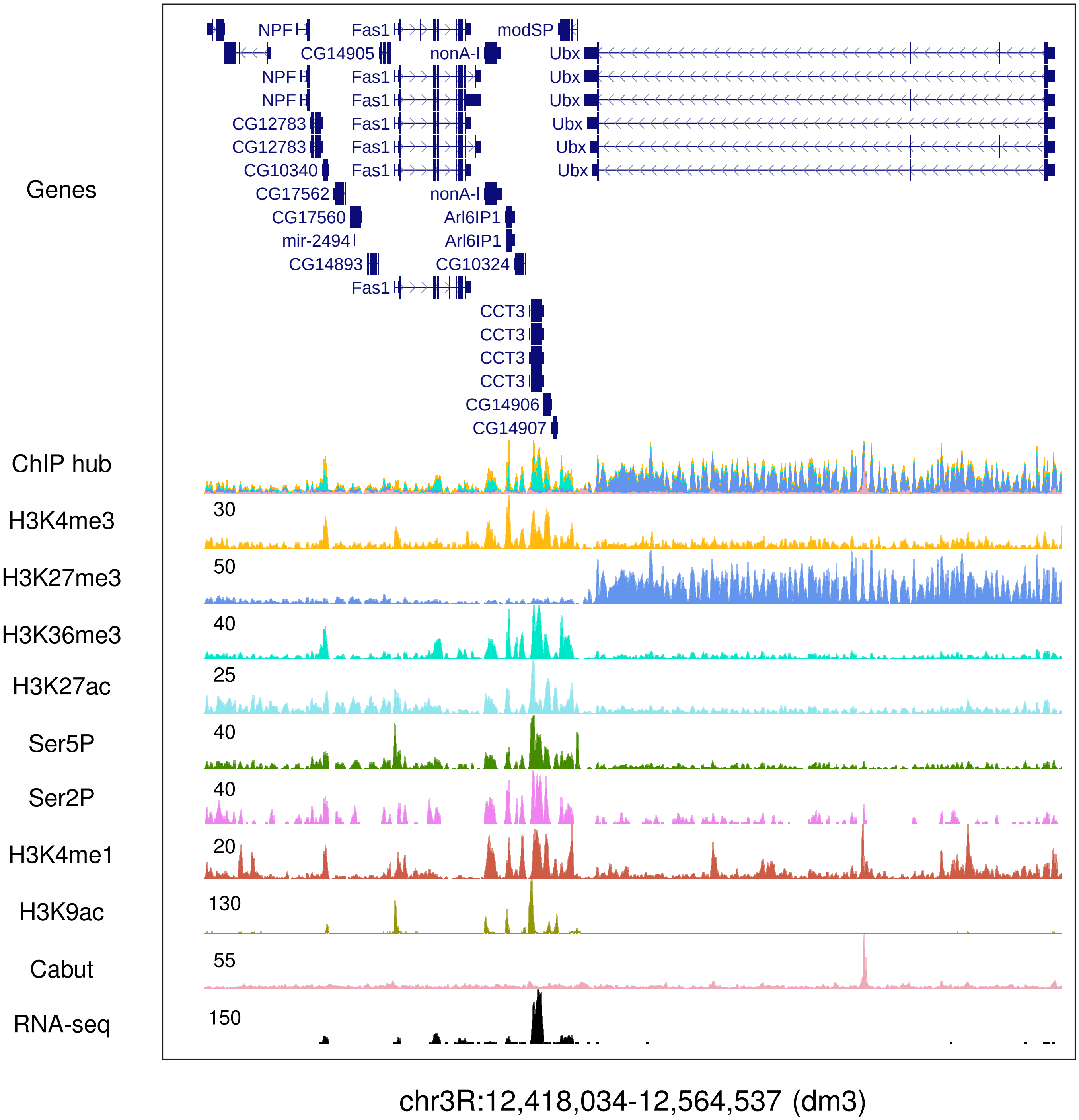

Suppl. Fig. 1.

SeqCode ChIP-seq and RNA-seq profiles from *Drosophila melanogaster* wing imaginal discs for visualization in genome browsers
