# Supplementary figures and images for: Productive visualization of high-throughput sequencing data using the SeqCode open portable platform
Source: Sci Rep. 2021 Oct 1;11:19545. doi: 10.1038/s41598-021-98889-7 (PMC8486768; doi:10.1038/s41598-021-98889-7)

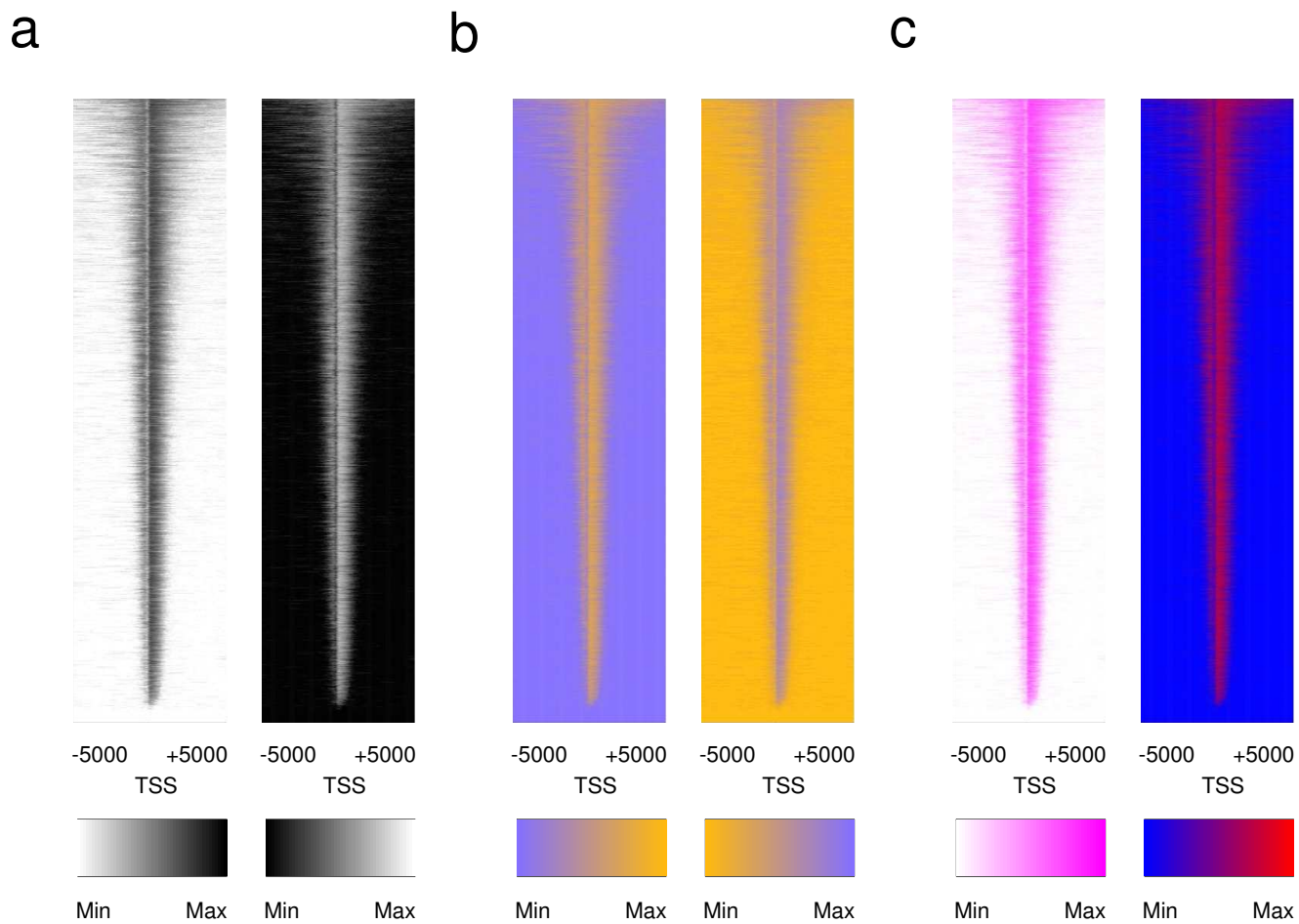

## Suppl. Fig. 2.

Multiple chromatic configurations of H3K4me3 in mESCs with SeqCode

Supplement: Supplementary file 2 — Supplementary Figure S2. [file 41598_2021_98889_MOESM2_ESM.pdf]

a

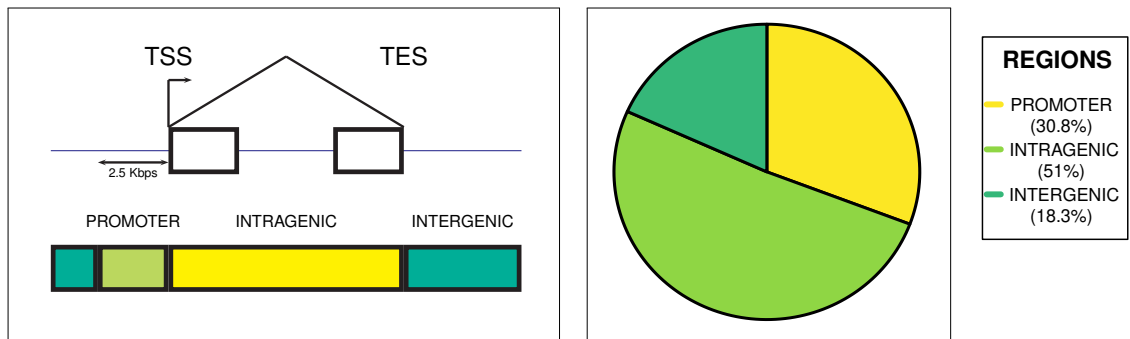

b

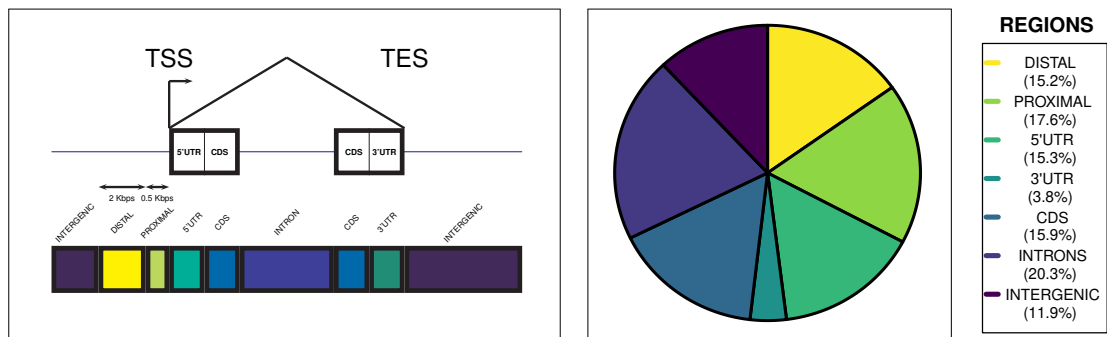

c

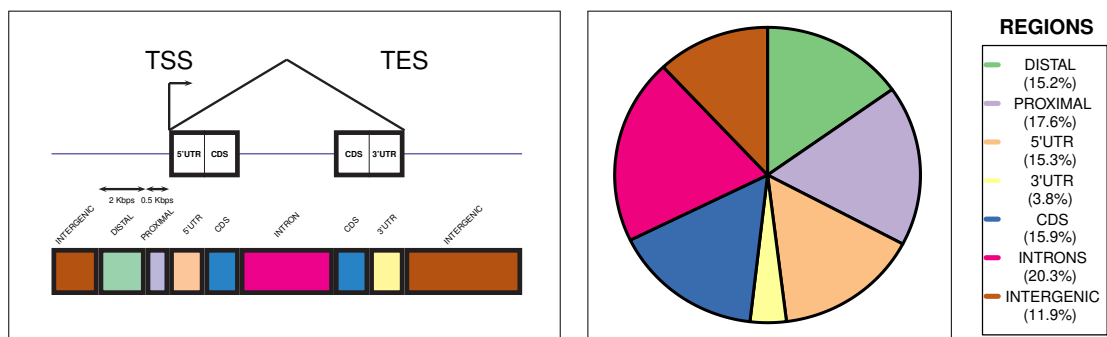

d

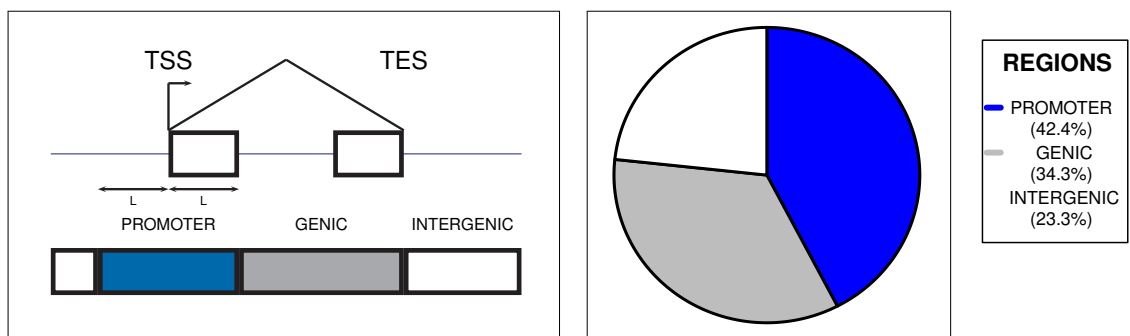

## Suppl. Fig. 3.

Configuration schemes of the genomeDistribution function

Supplement: Supplementary file 3 — Supplementary Figure S3. [file 41598_2021_98889_MOESM3_ESM.pdf]

a

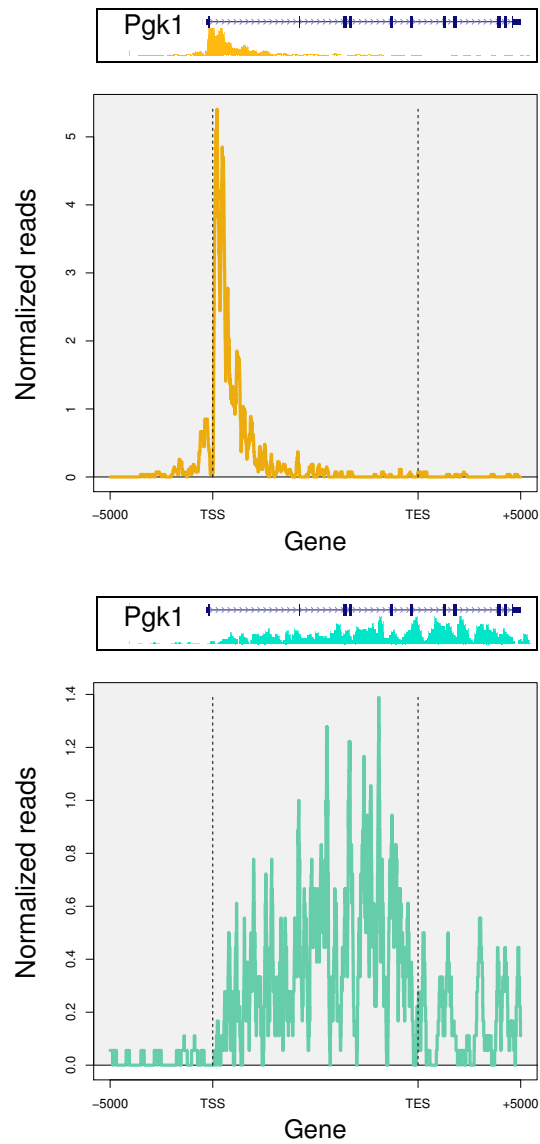

b

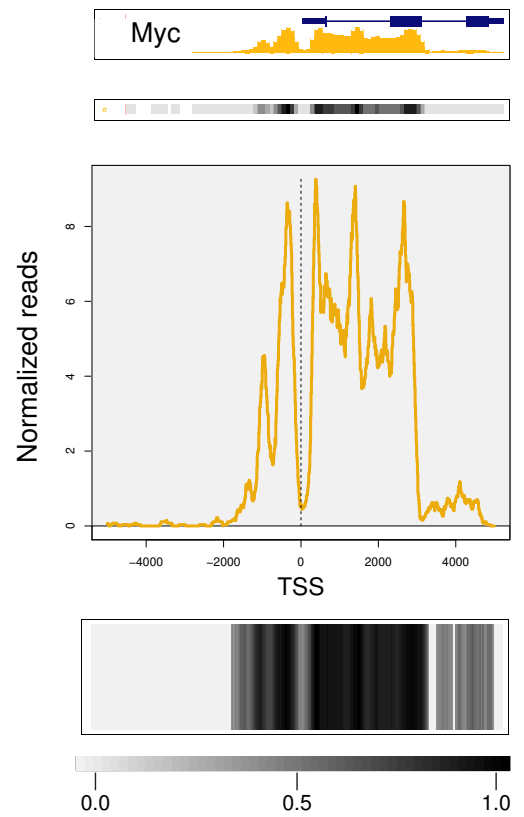

## Suppl. Fig. 4.

SeqCode average plots on individual genes in H3K4me3 and H3K36me3 from mESCs

Supplement: Supplementary file 4 — Supplementary Figure S4. [file 41598_2021_98889_MOESM4_ESM.pdf]

a

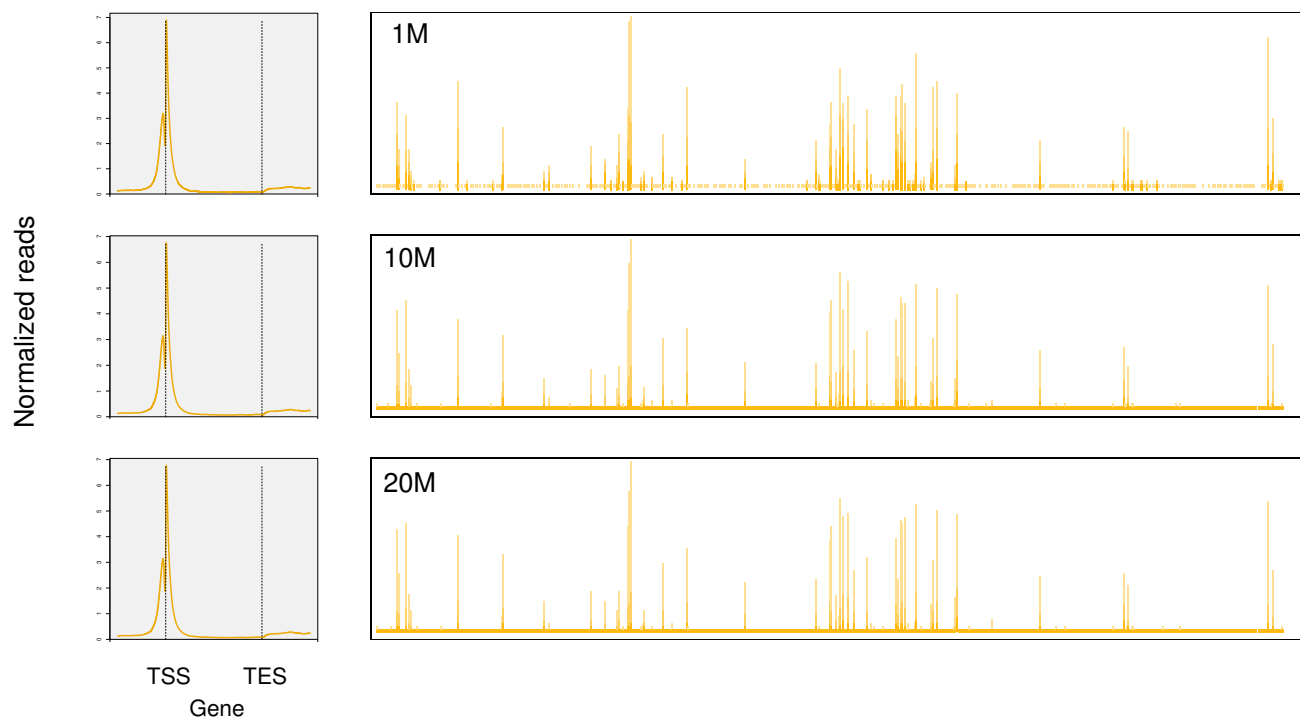

b

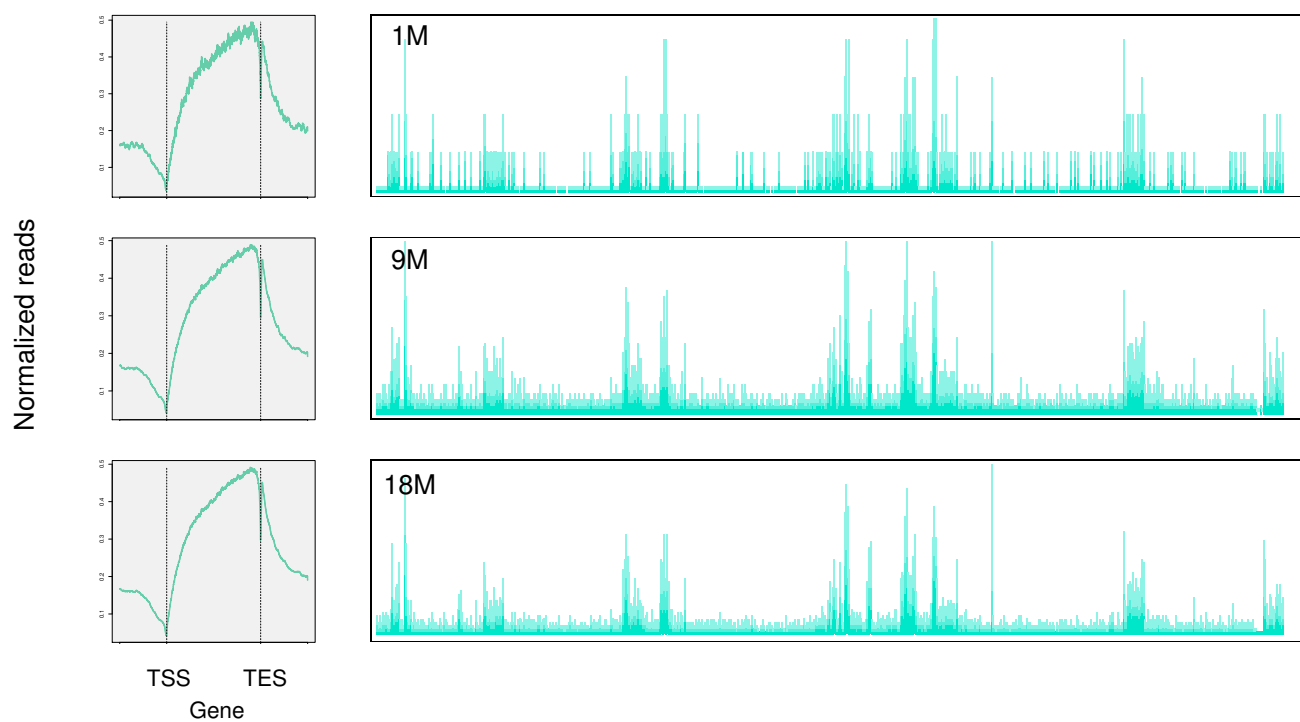

## Suppl. Fig. 5.

Visualization of mESC ChIP-seq profiles with different coverage in SeqCode

Supplement: Supplementary file 5 — Supplementary Figure S5. [file 41598_2021_98889_MOESM5_ESM.pdf]

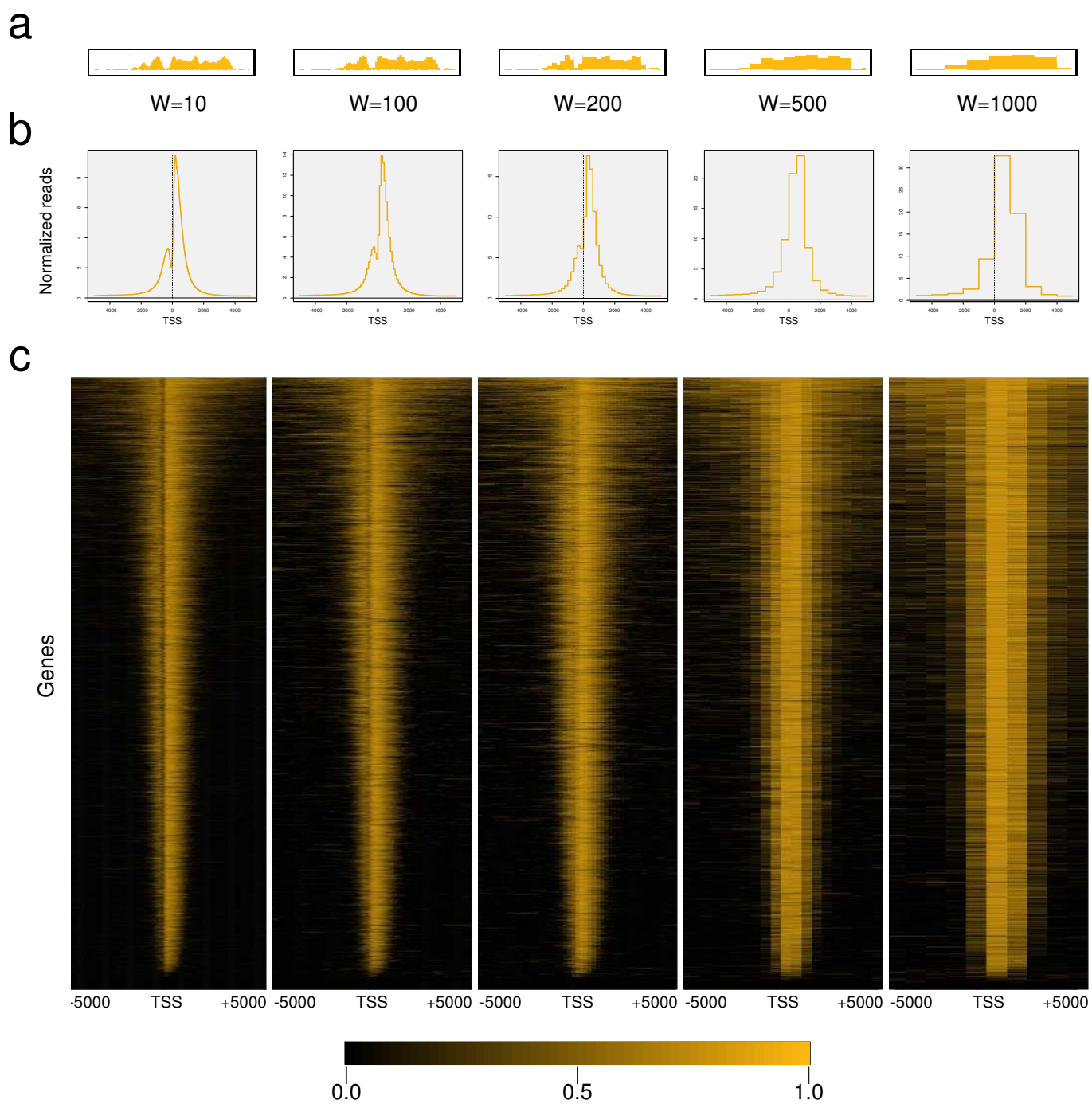

**Suppl. Fig. 6.**

Multiple graphical representations using different window size with SeqCode

Supplement: Supplementary file 6 — Supplementary Figure S2. [file 41598_2021_98889_MOESM6_ESM.pdf]

**a**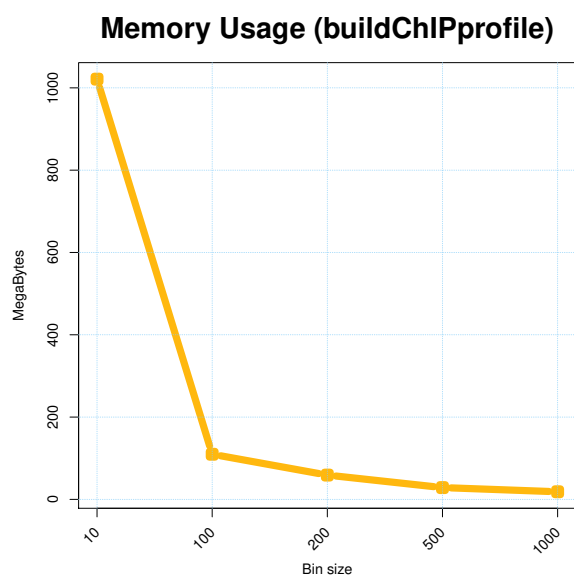**b**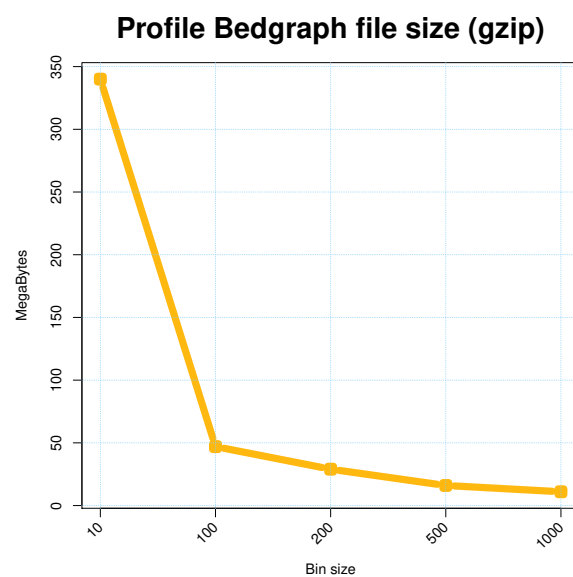**c**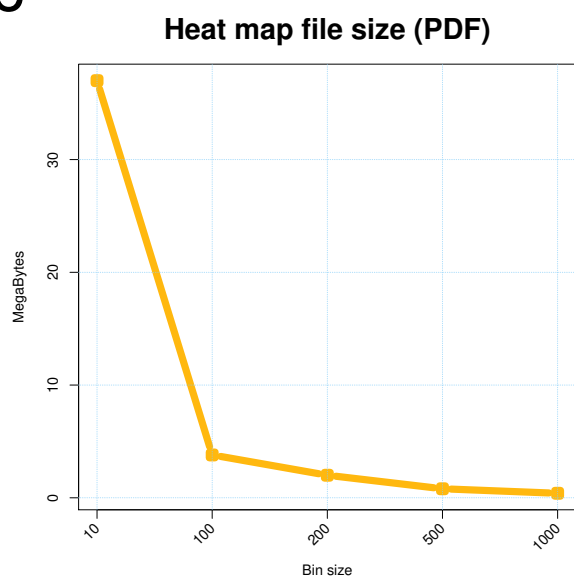**d**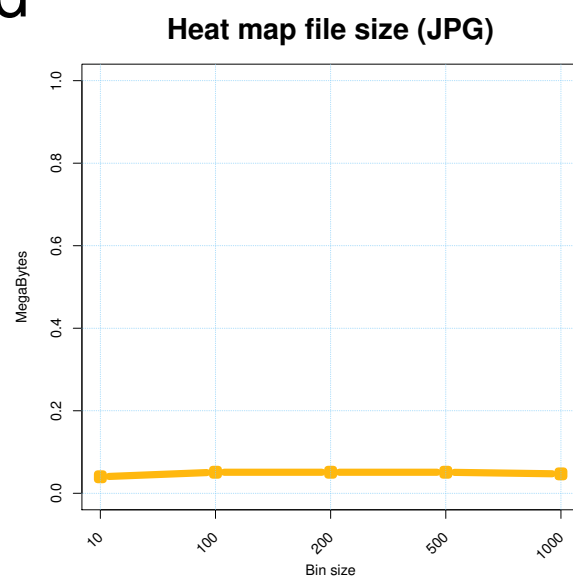

## Suppl. Fig. 7.

Computational requirements of SeqCode using different window size

Supplement: Supplementary file 7 — Supplementary Figure S7. [file 41598_2021_98889_MOESM7_ESM.pdf]

a

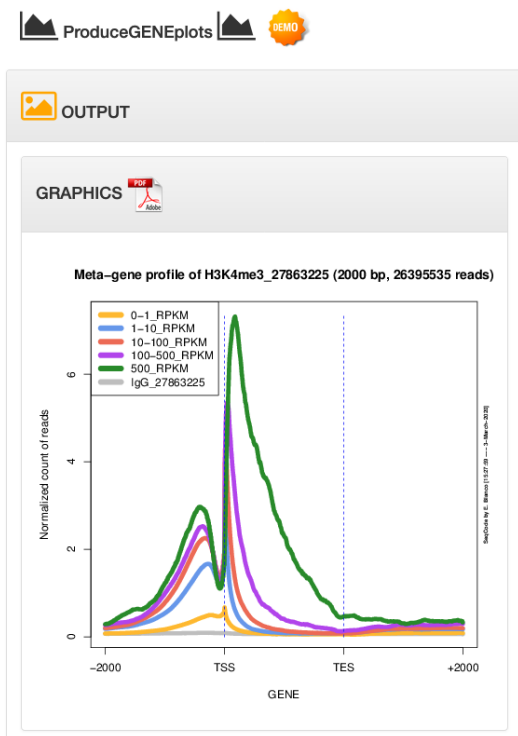

b

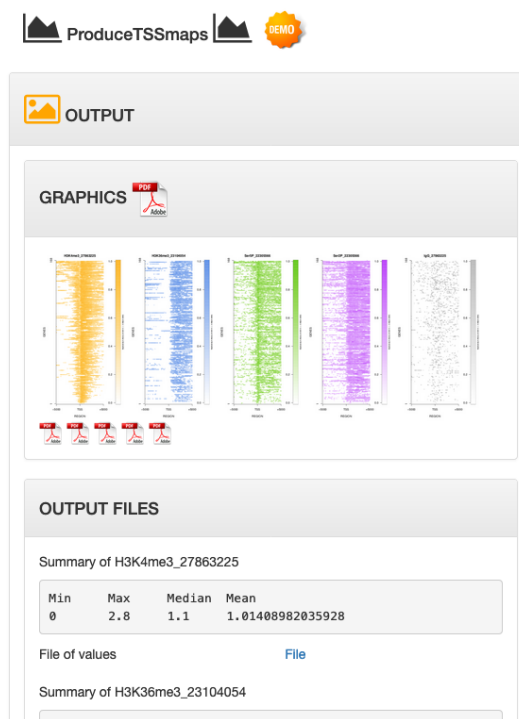

c

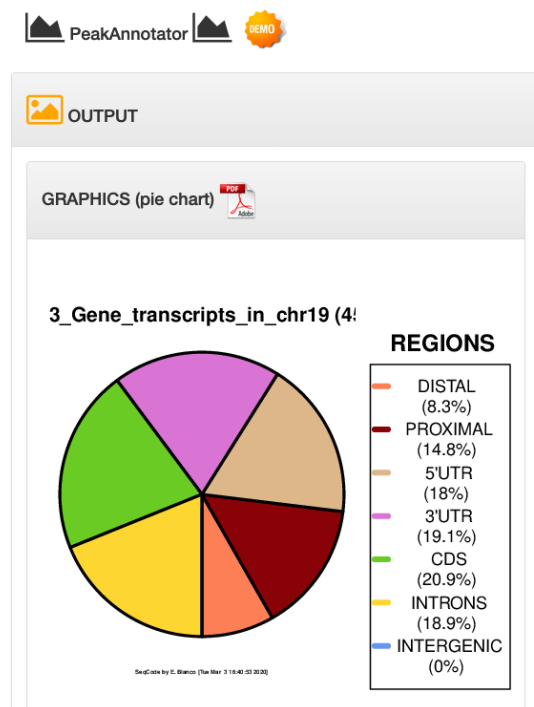

d

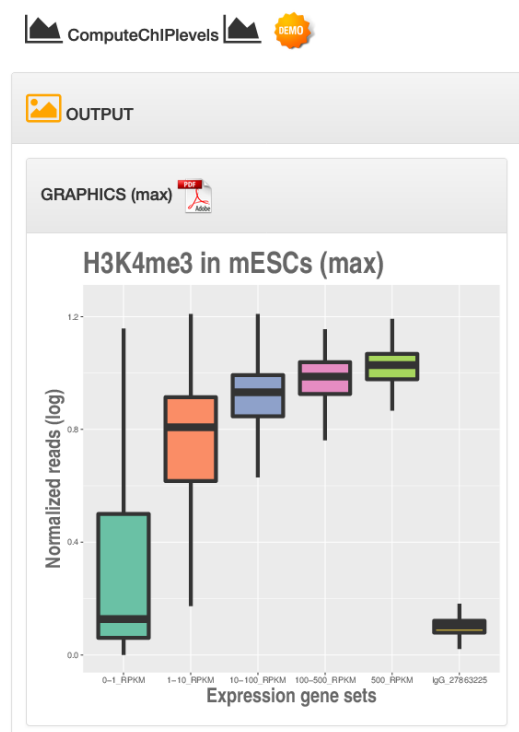

Suppl. Fig. 9.

NGS tools of the SeqCode web site

Supplement: Supplementary file 9 — Supplementary Figure S9. [file 41598_2021_98889_MOESM9_ESM.pdf]

a

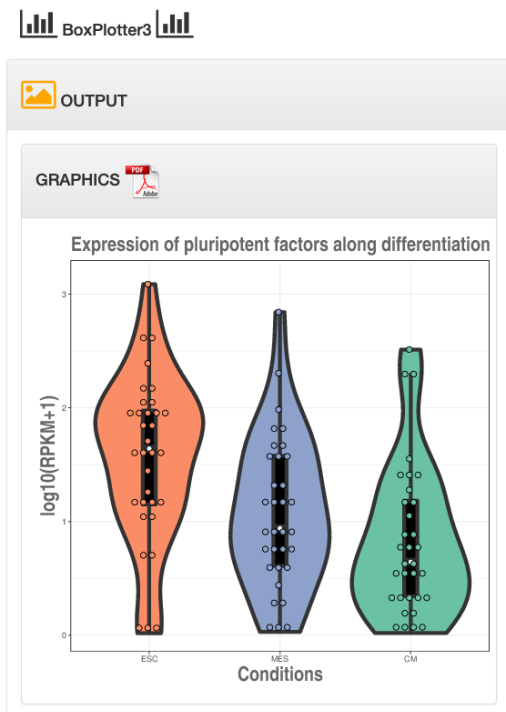

b

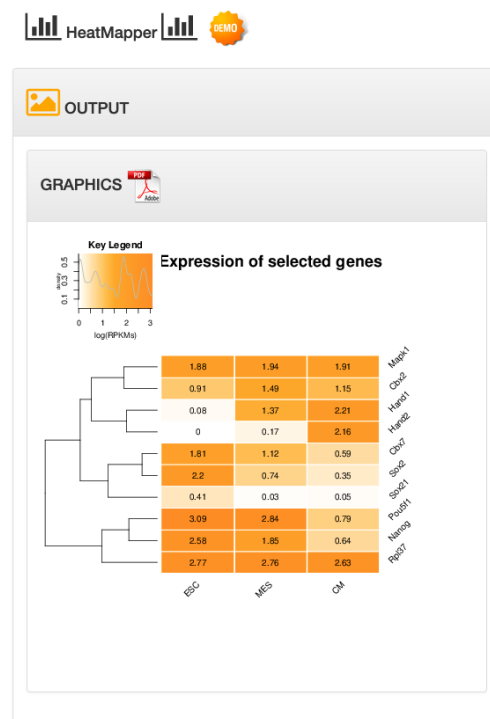

c

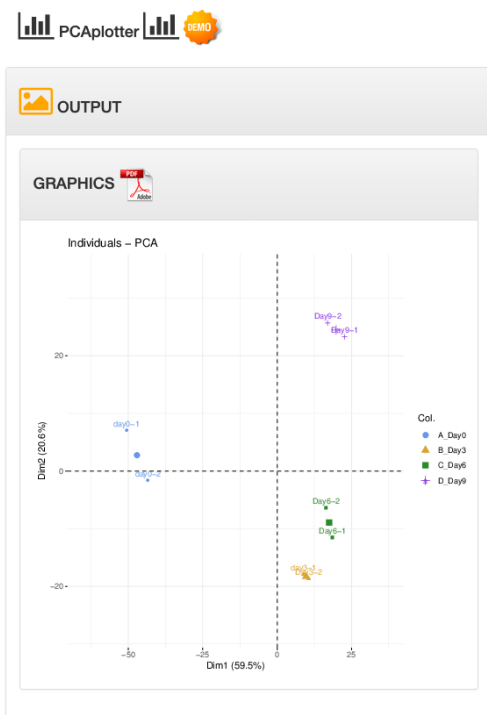

d

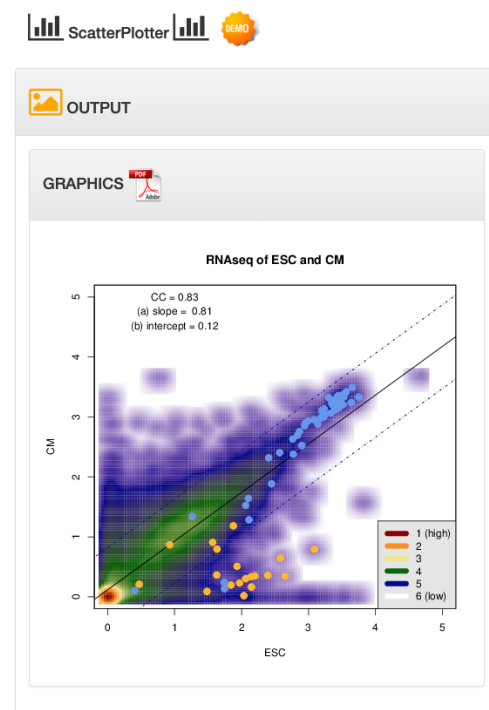

Suppl. Fig. 10.

Data Sets tools of the SeqCode web site

Supplement: Supplementary file 10 — Supplementary Figure S10. [file 41598_2021_98889_MOESM10_ESM.pdf]

a

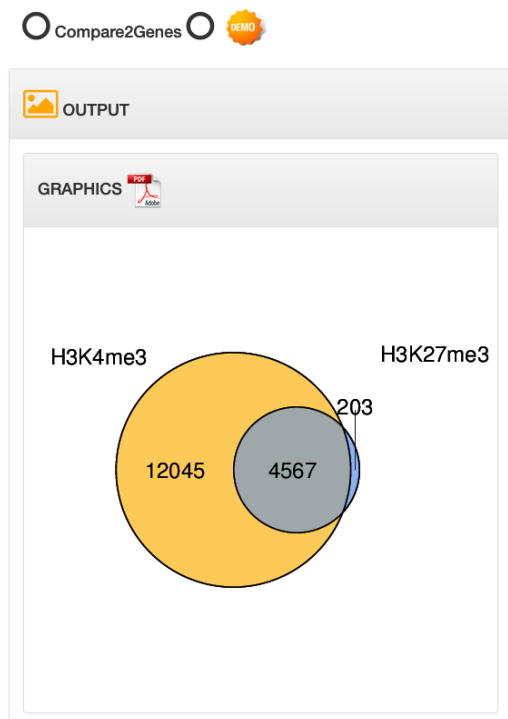

b

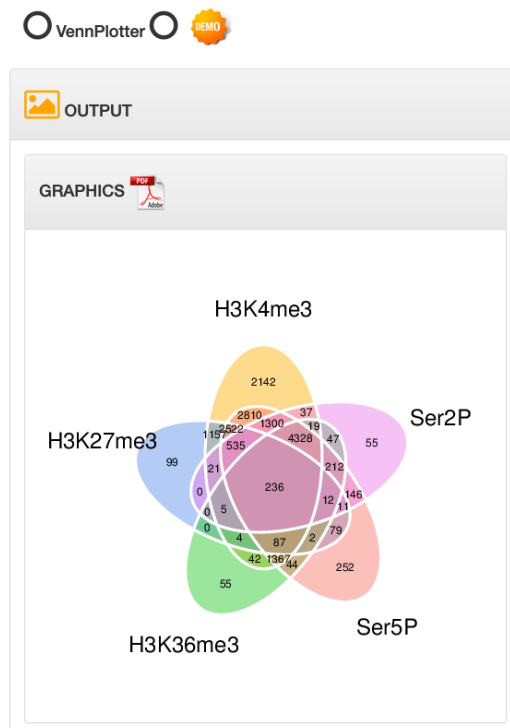

c

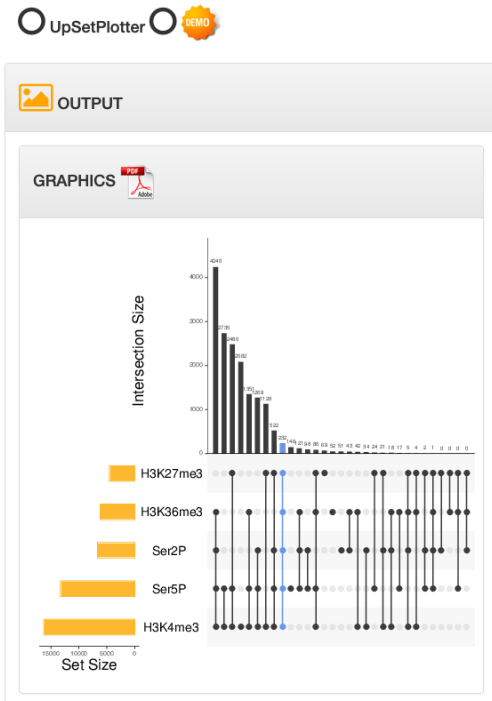

d

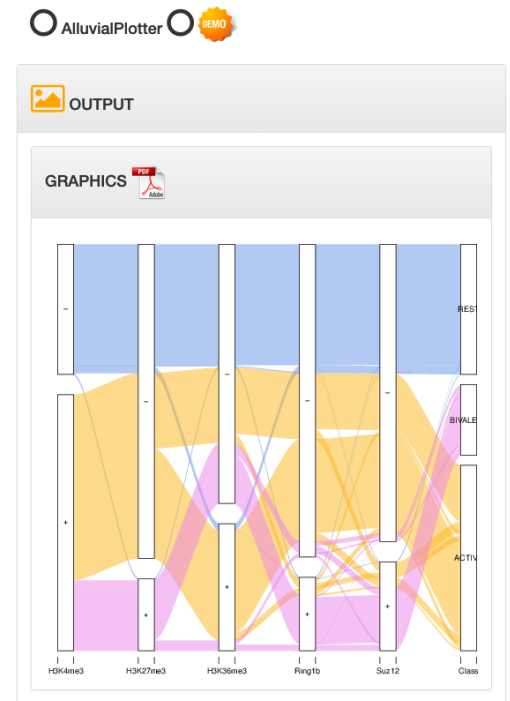

Suppl. Fig. 11.

Gene Sets tools of the SeqCode web site

Supplement: Supplementary file 11 — Supplementary Figure S11. [file 41598_2021_98889_MOESM11_ESM.pdf]

a

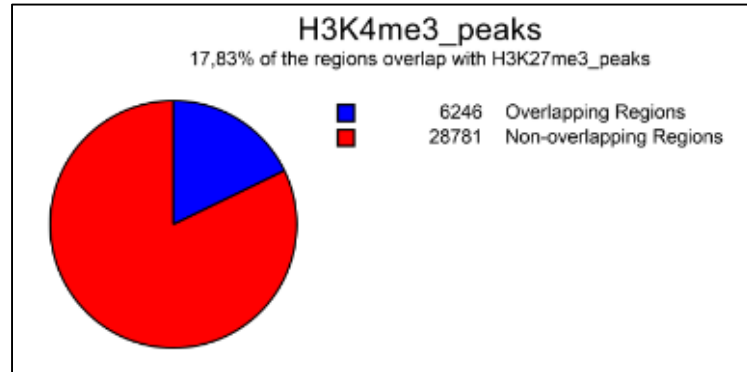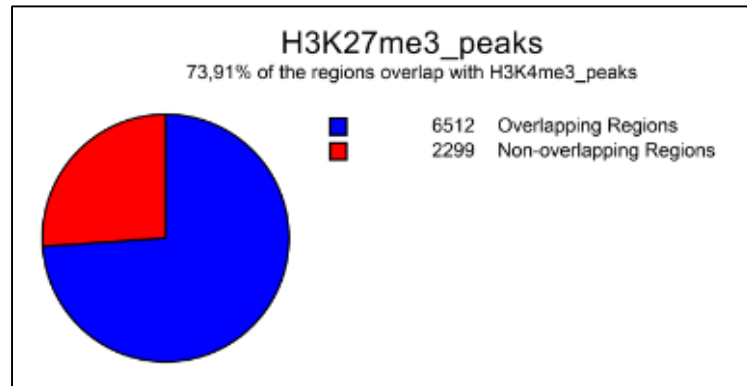

b

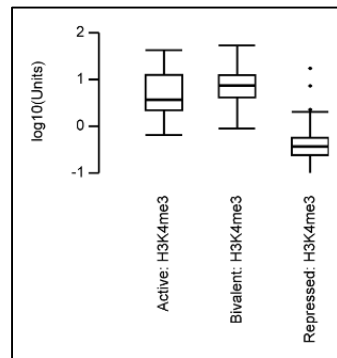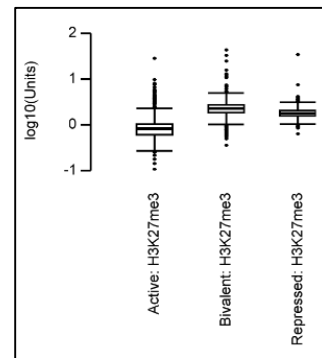

c

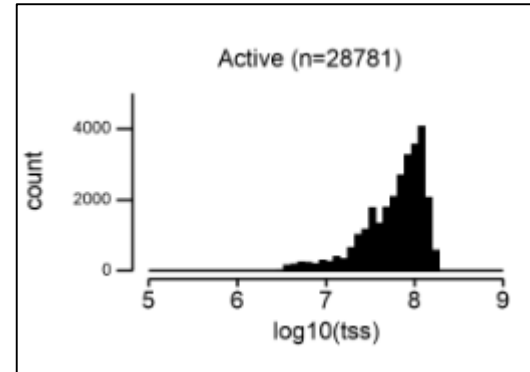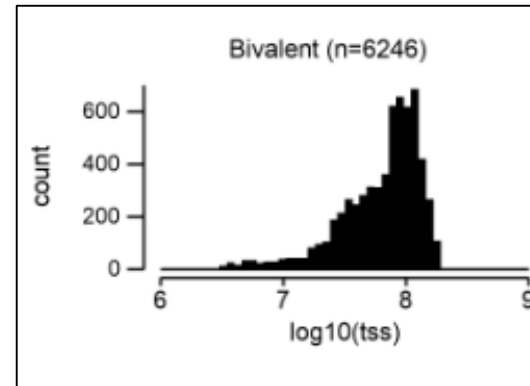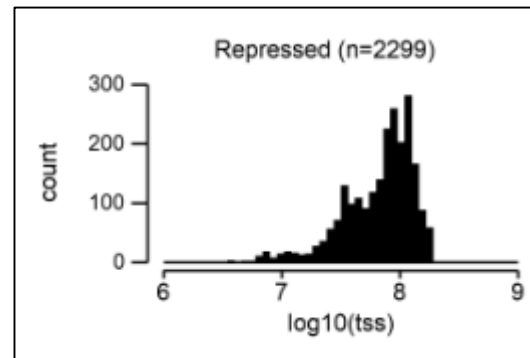

d

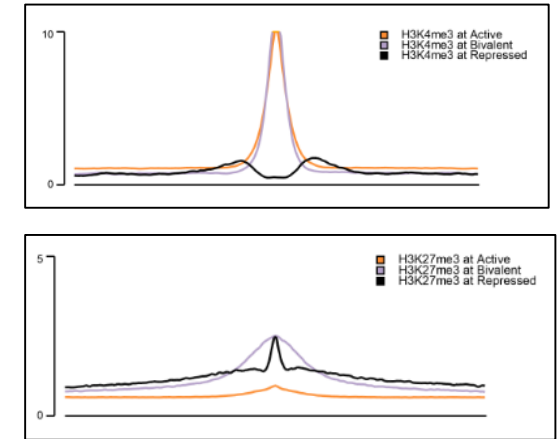

e

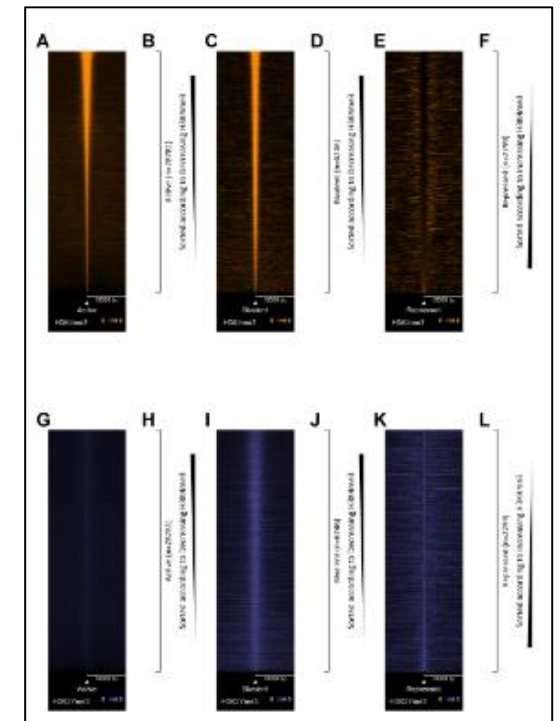

Suppl. Fig. 12. Output of the EaSeq software on the bivalency analysis of mESCs

Supplement: Supplementary file 12 — Supplementary Figure S12. [file 41598_2021_98889_MOESM12_ESM.pdf]

a

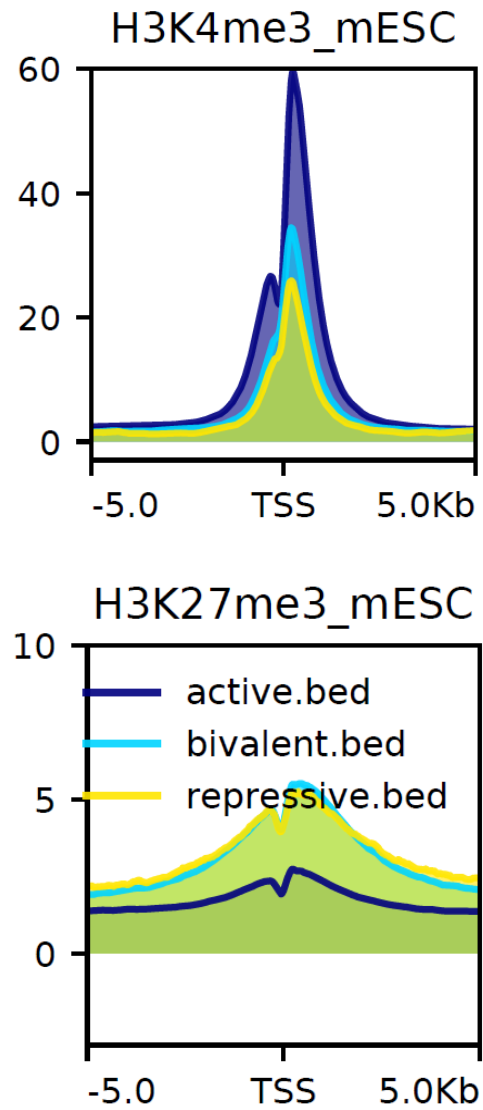

b

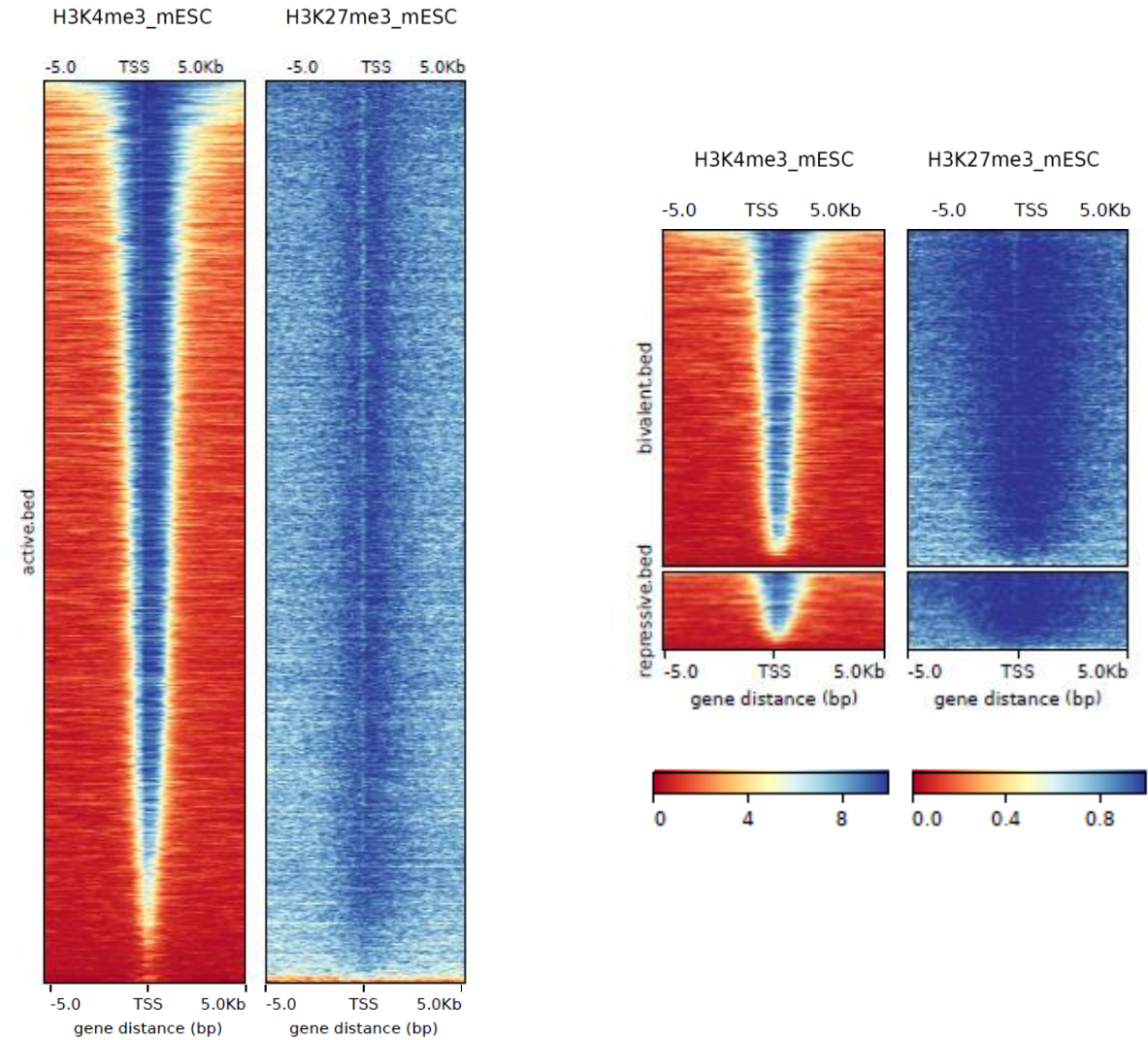

Suppl. Fig. 13. Output of the DeepTools2 software on the bivalency analysis of mESCs

Supplement: Supplementary file 13 — Supplementary Figure S13. [file 41598_2021_98889_MOESM13_ESM.pdf]

a

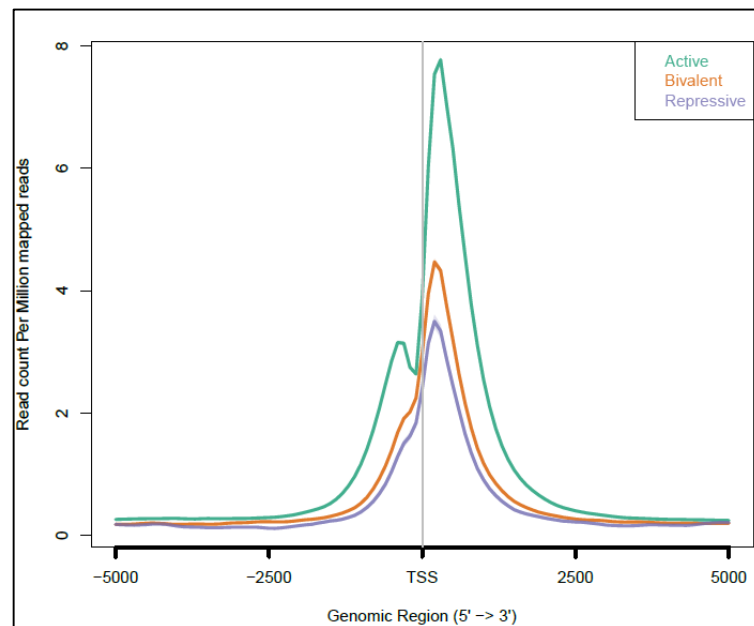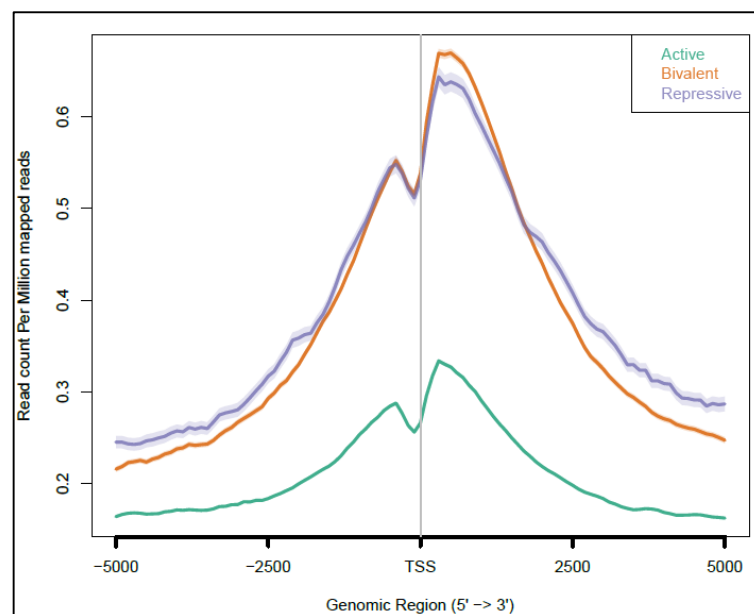

b

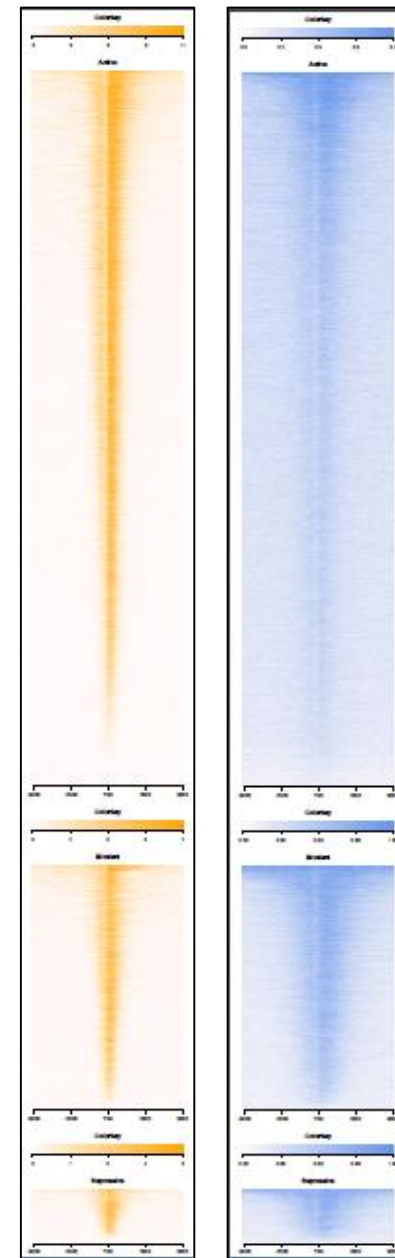

**Suppl. Fig. 14. Output of the ngs.plot software on the bivalency analysis of mESCs**

Supplement: Supplementary file 14 — Supplementary Figure S14. [file 41598_2021_98889_MOESM14_ESM.pdf]
