# Supplementary material for: Productive visualization of high-throughput sequencing data using the SeqCode open portable platform
Source: Sci Rep. 2021 Oct 1;11:19545. doi: 10.1038/s41598-021-98889-7 (PMC8486768; doi:10.1038/s41598-021-98889-7)
Supplement: Supplementary file 8 — Supplementary Figure S8. [file 41598_2021_98889_MOESM8_ESM.pdf]

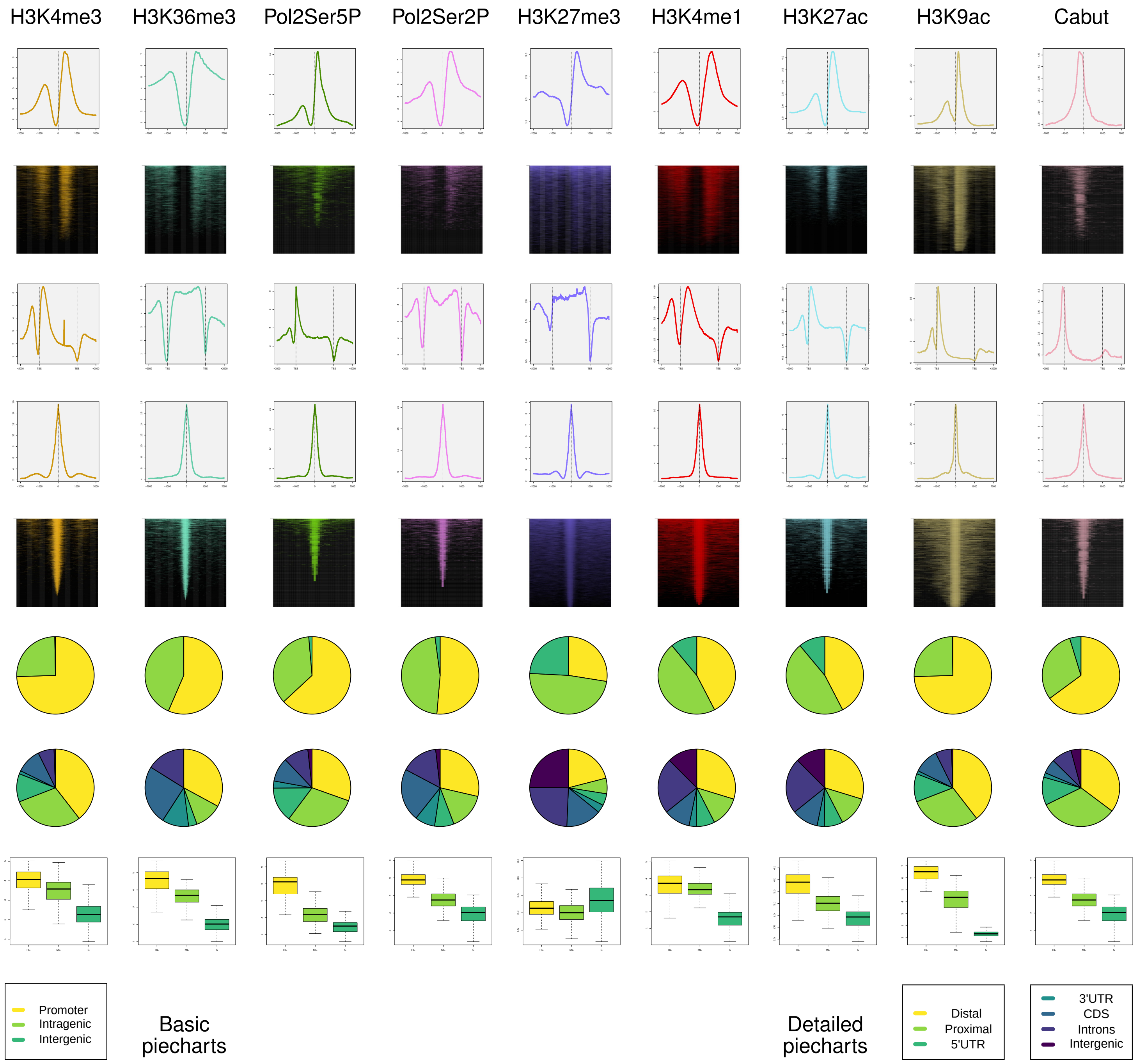

Suppl. Fig. 8.

Panel of epigenetic markers and the Cabut transcription factor in *Drosophila melanogaster* wing imaginal discs generated by SeqCode
