# Supplementary material for: Productive visualization of high-throughput sequencing data using the SeqCode open portable platform
Source: Sci Rep. 2021 Oct 1;11:19545. doi: 10.1038/s41598-021-98889-7 (PMC8486768; doi:10.1038/s41598-021-98889-7)
Supplement: Supplementary file 16 — Supplementary Legends. [file 41598_2021_98889_MOESM16_ESM.docx]

**Supplementary Legends**

**Supp. Fig. 1. SeqCode ChIP-seq and RNA-seq profiles from *Drosophila melanogaster* wing imaginal discs for visualization in genome browsers.** Region repressed for transcription by PcG proteins containing the *Ubx* gene of the Bithorax complex (BX-C). Raw data were retrieved from ^1-3^. The SeqCode buildChIPprofile function (window size = 100) was used to generate each custom track from resulting BAM files. Composite ChIP-seq supertrack integrates all the information of each individual track shown below.

**Supp. Fig. 2. Multiple chromatic configurations of H3K4me3 in mESCs with SeqCode.** (a–c) The same heatmap of the ChIP-seq signal around the TSS of H3K4me3 target genes generated with the produceTSSmaps function is shown using a different color scheme on each case: (a) black and white, (b) yellow and blue, and (c) magenta/white and red/blue. Raw data were retrieved from ^4^.

**Supp. Fig. 3. Configuration schemes of the genomeDistribution function.** (a–c) The graphical summary is shown in three modes (a, default; b, detailed using R package viridis palette; c, detailed using accent palette from ColorBrewer (https://colorbrewer2.org/#type=qualitative&scheme=Accent&n=7); and d, compact) for the ChIP-seq peaks reported for H3K4me3 in mESCs^5^. For each representation mode, a pictogram is included of the genomic regions reported (on the left) and the resulting genome distribution (on the right).

**Supp. Fig. 4. SeqCode average plots on individual genes in H3K4me3 and H3K36me3, from mESCs.** (a) *Pgk1* gene profile screenshot and average distribution of the ChIP-seq signals of both marks along the gene body. (b) *Myc* gene profile screenshot, heatmap screenshot, SeqCode average distribution of the ChIP-seq signal, and SeqCode heatmap of the ChIP-seq signal around the TSS for H3K4me3. The following SeqCode functions were used: buildChIPprofile, produceGENEplots, produceTSSplots and produceTSSmaps. Raw data were retrieved from ^4,6^.

**Supp. Fig. 5. Visualization of mESC ChIP-seq profiles with different coverage in SeqCode.** (a and b) Using different sequencing depths for H3K4me3 (a: 1, 10, and 20 million reads) and H3K36me3 (b: 1, 9, and 18 million reads) in mESCs, the average distribution of the ChIP-seq signal is displayed along the gene body of target genes calculated with the produceGENEplots function (left), and of the SeqCode profile generated by the buildChIPprofile function as visualized in the UCSC genome browser (right) ^7^. Raw data were retrieved from ^4,6^.

**Supp. Fig. 6. Multiple graphical representations using different window size with SeqCode.** (a–c) Information about H3K4me3 in mESCs provided for each configuration (from left to right, high to low resolution): (a) genome browser screenshot of the custom track (buildChIPprofile), (b) average distributions (produceTSSplots), and (c) heatmaps (produceTSSmaps) of the ChIP-seq signal around the TSS of H3K4me3 target genes. Raw data were retrieved from ^4^.

**Supp. Fig. 7. Computational requirements of SeqCode using different genome bin size.** (a and b) Amount of RAM necessary to build the custom track of the ChIP-seq of H3K4me3 in mESCs, and hard drive space required to store the resulting custom track in BedGraph format. (c and d) Hard drive space required to store the resulting heatmap of the ChIP-seq of H3K4me3 in mESCs, and the same value after converting the PDF plot into a bitmap archive. Raw data were retrieved from ^4^.

**Supp. Fig. 8. Panel of epigenetic markers and the Cabut transcription factor in *Drosophila melanogaster* wing imaginal discs generated by SeqCode.** For each ChIP-seq signal and the corresponding set of target genes, the average distribution (produceTSSplots) and heatmap (produceTSSmaps) around the TSS, average distribution along the gene body (produceGENEplots), the average distribution (producePEAKplots) and heatmap (producePEAKmaps) around the center of the peaks, the genomic distribution of each set of peaks (genomeDistribution), and the ChIP-seq levels normalized by the total number of reads (recoverChIPlevels) are shown for 100 highly expressed genes (more than 1000 RPKMs; red), 100 moderately expressed genes (100-500 RPKMs; yellow), and 100 silenced genes (0–1 RPKMs; blue). Raw data were retrieved from ^1-3^. Target genes of each ChIP-seq were identified with the matchpeaksgenes routine.

**Supp. Fig. 9. NGS tools of the SeqCode web site.** (a–d) Screenshots of several tutorials of this family of services. (a) The produceGENEplots function generates the meta-gene plot of the ChIP-seq of H3K4me3 in mESCs for five lists of genes ranked by expression (RPKMs) together with the profile of the control sample (IgG). (b) The produceTSSmaps function builds the heatmap of five ChIP-seq experiments in mESCs (H3K4me3, H3K36me3, RNA polymerase II Ser5P, RNA polymerase II Ser2P, and IgG) for a selection of expressed genes. (c) Pie chart of distribution of genomic features calculated with the genomeDistribution service for RefSeq transcripts annotated in chr19 (mouse assembly mm9). (d) Maximum number of normalized reads over promoters of five lists of genes ranked by expression (RPKMs) together with the values in the control sample (IgG) as calculated by the recoverChIPlevels function.

**Supp. Fig. 10. Dataset tools of the SeqCode website.** (a–d) Screenshots of several tutorials of this family of services. (a) Violin plots depicted with the Boxplotter3 function showing the expression in RPKMs of genes involved in pluripotency along three time-points of differentiation (MES, mesodermal precursors; CM, cardiomyocytes). (b) Heatmap of expression of selected genes along the same differentiation timeline done with the HeatMapper tool. (c) PCA analysis performed by the PCAplotter function of the expression data about differentiation of embryoid bodies (EBs) along four time-points^8^. (d) Scatterplot of gene expression generated with the ScatterPlotter application, showing the correlation between ESCs and CMs for the whole set of genes in the mouse genome^9^.

**Supp. Fig. 11. Geneset tools of the SeqCode website.** (a–d) Screenshots of several tutorials of this family of services. (a) Overlap between the target genes of H3K4me3 and H3K27me3 in mESCs performed by the Compare2Genes application. (b) Overlap between the target genes of H3K4me3, H3K27me3, H3K36me3, and RNA polymerase II Ser2P and Ser5P in mESCs, calculated with the VennPlotter command. (c) UpSet plot^10^ generated with the UpSetPlotter function over the same lists of genes. (d) Alluvial diagram done with AlluvialPlotter depicting the transitions between target genes of H3K4me3, H3K27me3, H3K36me3, Ring1B, and Suz12 in mESCs. Genes in the genome were previously classified as active (marked only with H3K4me3, in orange), bivalent (H3K4me3 and H3K27me3, in violet) and the rest (no histone marks, in blue).

**Supp. Fig. 12. Output of the EaSeq software on the bivalency analysis of mESCs.** (a) Overlap of the two sets of H3K4me3 and H3K27me3 peaks identified in mESCs, performed with the function Overlap (Regionsets). (b) Signal strength of H3K4me3 and H3K27me3 ChIP-seq samples calculated with the function Quantify (Regionset and Dataset) over the three sets of peaks (active, bivalent, repressive) defined in Figure 6. (c) Distribution of distances of each class of peaks to RefSeq gene transcripts calculated with the function Annotate (Regionset and Geneset). (d) Aggregated plots of H3K4me3 and H3K27me3 ChIP-seq samples over the three peak sets described above generated with the functions Average and Overlay (Dataset and Regionset). (e) Heatmaps of H3K4me3 and H3K27me3 ChIP-seq experiments centered on the peaks of active, bivalent, and repressed peaks generated with the function HeatMap (Regionset and Dataset).

**Supp. Fig. 13. Output of the DeepTools2 software on the bivalency analysis of mESCs.** (a) Aggregated plots of H3K4me3 and H3K27me3 ChIP-seq samples centered over the TSSs of the three sets of genes described in Figure 6 (active, bivalent, and repressive) generated with the function plotProfile. (b) Heatmaps of H3K4me3 and H3K27me3 ChIP-seq samples centered over the TSSs of the three sets of genes generated with the function plotHeatmap. In both cases, the BAM files of aligned reads were previously processed with the bamCoverage routine to generate bigWig files, and the computeMatrix (reference-point) function was executed to calculate the matrix of values for plotting. Gene lists were transformed in BED files of genomic coordinates using GAWK in command line, according to RefSeq annotations.

**Supp. Fig. 14. Output of the ngs.plot software on the bivalency analysis of mESCs.** (a) Aggregated plots of H3K4me3 and H3K27me3 ChIP-seq samples (top and bottom, respectively) centered over the TSSs of the three sets of genes described in Figure 6 (active, bivalent, and repressive). (b) Heatmaps of H3K4me3 and H3K27me3 ChIP-seq samples (in orange and blue, respectively) centered over the TSSs of the three sets of genes described above. The ngs.plot.r function was executed to generate metaplots and heatmaps in a single run, using a configuration file that provides the location of BAM files and the lists of genes.

**Suppl. Fig. 15. Guidelines of selection of alternative visualizations depending on the biological question.** (a) How to select the appropriate visualization mode when analyzing one single sequencing sample. Q1: Once a new sample has been sequenced, the result of the mapping can be imported into a genome browser to inspect the quality of the profile prior to further analysis; Q2: Heatmaps and meta-plots are particularly useful to elucidate whether a particular element tends to recognize sharp or broad regions in the genome; Q3: To reveal a particular binding preference, genome pie charts and meta-plots/heatmaps centered along a particular viewpoint (*e.g.* TSS, gene body) characterize a distribution of sequencing peaks across all categories of genomic elements; Q4: To identify the putative association of the biological feature that has been sequenced with a particular group of genomic elements (genes, enhancers, etc.), signal strength at every subset of the full collection of items can be visualized in boxplots. Heatmaps and meta-plots are also useful to graphically represent such differences among groups; Q5: After peak calling, sequencing peaks in the vicinity of genomic features annotated by an external consortium (e.g. RefSeq) can be utilized to build a list of targets (genes, enhancers) for ontology analyses. (b) How to select the appropriate visualization mode when analyzing two or more sequencing samples (*i.e.* replicates, distinct experimental conditions). Q6: Contrast of multiple sequencing experiments in the genome browser over the same region (*i.e.* the gene of interest) facilitates the interpretation of differences/similarities in the occupancy patterns; Q7: Quantitative differences on selected genomic intervals (genomic bins, genes, promoters, enhancers, etc.) among samples are statistically studied using boxplots. Moreover, signal strength from multiple experiments can be graphically depicted with meta-plots of overlapping lines, heat-maps of multiple columns (one column per experiment) and pairwise scatterplots; Q8: Changes between samples in the distribution of preferences for any particular genomic element (e.g. promoter, gene, CDS, intergenic, etc.) can be highlighted inside meta-plots and heatmaps with selected viewpoints, or with the comparison of distinct pie charts of genome composition using spie charts; Q9: For each pair of samples, the subtraction of the signal in the first experiment from the signal in the second one can be very informative to identify enrichments in any direction; Q10: Two or more lists of putative target genes of the sequencing experiments can be compared with Venn diagrams and UpSet charts in order to assess their similarity. On the other hand, matching operations to evaluate the similarity of lists of genomic intervals can be performed and the overlap between records can be visualized in genomic browsers.

**References**

1 Perez-Lluch, S. *et al.* Genome-wide chromatin occupancy analysis reveals a role for ASH2 in transcriptional pausing. *Nucleic Acids Res* **39**, 4628-4639, doi:10.1093/nar/gkq1322 (2011).

2 Perez-Lluch, S. *et al.* Absence of canonical marks of active chromatin in developmentally regulated genes. *Nat Genet* **47**, 1158-1167, doi:10.1038/ng.3381 (2015).

3 Ruiz-Romero, M., Blanco, E., Paricio, N., Serras, F. & Corominas, M. Cabut/dTIEG associates with the transcription factor Yorkie for growth control. *EMBO Rep* **16**, 362-369, doi:10.15252/embr.201439193 (2015).

4 Tee, W. W., Shen, S. S., Oksuz, O., Narendra, V. & Reinberg, D. Erk1/2 activity promotes chromatin features and RNAPII phosphorylation at developmental promoters in mouse ESCs. *Cell* **156**, 678-690, doi:10.1016/j.cell.2014.01.009 (2014).

5 Beringer, M. *et al.* EPOP Functionally Links Elongin and Polycomb in Pluripotent Stem Cells. *Mol Cell* **64**, 645-658, doi:10.1016/j.molcel.2016.10.018 (2016).

6 Ballare, C. *et al.* Phf19 links methylated Lys36 of histone H3 to regulation of Polycomb activity. *Nat Struct Mol Biol* **19**, 1257-1265, doi:10.1038/nsmb.2434 (2012).

7 Haeussler, M. *et al.* The UCSC Genome Browser database: 2019 update. *Nucleic Acids Res* **47**, D853-D858, doi:10.1093/nar/gky1095 (2019).

8 Mas, G. *et al.* Promoter bivalency favors an open chromatin architecture in embryonic stem cells. *Nat Genet* **50**, 1452-1462, doi:10.1038/s41588-018-0218-5 (2018).

9 Morey, L. *et al.* Polycomb Regulates Mesoderm Cell Fate-Specification in Embryonic Stem Cells through Activation and Repression Mechanisms. *Cell Stem Cell* **17**, 300-315, doi:10.1016/j.stem.2015.08.009 (2015).

10 Conway, J. R., Lex, A. & Gehlenborg, N. UpSetR: an R package for the visualization of intersecting sets and their properties. *Bioinformatics* **33**, 2938-2940, doi:10.1093/bioinformatics/btx364 (2017).
